# A β-carboline natural product induces oxidative stress in *Fusarium graminearum* and limits the virulence of multiple fungal phytopathogens

**DOI:** 10.1128/mbio.01291-26

**Published:** 2026-07-10

**Authors:** Bradley Laflamme, Christopher Blackman, Lauren Ryder, Mackenzie E. W. Loranger, Weibin Ma, Yunjin Lee, Margaret Balcerzak, Jessie MacAlpine, Luke Whitesell, Nicholas J. Talbot, Keiko Yoshioka, Rajagopal Subramaniam, Nicole Robbins, Leah E. Cowen

**Affiliations:** 1 Department of Molecular Genetics, University of Toronto 204248, Toronto, Ontario, Canada; 2 Agriculture and Agri-Food Canada 6337, Ottawa, Ontario, Canada; 3 The Sainsbury Laboratory, University of East Anglia, Norwich Research Park 6106, Norwich, United Kingdom; 4 Department of Cell and Systems Biology, University of Toronto 98684, Toronto, Ontario, Canada; 5 Fungal Pathogenesis Section, Laboratory of Clinical Immunology and Microbiology, National Institute of Allergy & Infectious Diseases, National Institutes of Health 573242, Bethesda, Maryland, USA; Instituto Carlos Chagas, Curitiba, Brazil

**Keywords:** *Fusarium graminearum*, β-carbolines, antifungal agents, fungicide, phytopathogens, reactive oxygen species, strobilurins

## Abstract

The emergence of fungicide-resistant pathogens of many agronomically important crop species necessitates the development of novel strategies for controlling diseases caused by fungi. β-carbolines encompass a wealth of natural products with diverse modes of action that lead to a myriad of phenotypic effects. While these molecules have been previously described as having antifungal activity against select species, the potential of β-carbolines to limit agricultural diseases caused by fungi has not been fully explored. Here, we report that several β-carbolines strongly inhibit growth and conidial germination in the devastating cereal pathogen *Fusarium graminearum*. We prioritized 1-carboxylic acid β-carboline (1-CABC) for downstream functional analyses, finding that, unlike the antioxidant properties of other, more commonly studied β-carbolines, 1-CABC induces oxidative stress and results in a loss of mitochondrial membrane potential. This bioactivity leads to a strong synergistic interaction with mitochondrial complex III inhibitors, including strobilurins, with the compound combination enhancing the induction of reactive oxygen species. We also showed that 1-CABC is effective as a single agent and in combination with strobilurins in models of infection for three fungal phytopathogens: *F. graminearum*, *Botrytis cinerea*, and *Magnaporthe oryzae*. Finally, we evaluated the effects of 1-CABC against a panel of diverse human fungal pathogens (encompassing both yeasts and molds). While 1-CABC antifungal activity is largely conserved across all fungi tested, the underlying mechanism(s) governing this bioactivity appear distinct. Collectively, these data strongly support future, more detailed mechanistic studies of 1-CABC against a range of fungal pathogens.

**IMPORTANCE:**

Fungal diseases are a major threat to global food production, and many crop pathogens are becoming resistant to existing fungicides. This study identifies a promising new strategy to combat these infections using a class of molecules called β-carbolines. Here, we found that one compound in particular, 1-carboxylic acid β-carboline (1-CABC), strongly blocks the growth of several destructive fungal pathogens, including *Fusarium graminearum*, a devastating pathogen of wheat. Unlike related compounds, 1-CABC increased the production of oxidative stress inside the pathogen, and importantly, the compound worked even better when combined with fungicides that inhibit mitochondrial function. The treatment was successful against multiple plant pathogens in infection models of plants, highlighting its potential for agricultural use. The study also showed activity against human fungal pathogens, although the mechanism responsible for this bioactivity appears distinct. Overall, this work highlights new antifungal strategies to protect crops, with the ultimate goal of improving food security.

## INTRODUCTION

Fungal phytopathogens pose a growing threat to global food security, both due to damage to crops and production of carcinogenic mycotoxins, with innate and acquired resistance to established fungicides exacerbating the problem (1, 2). The limited protection currently offered by fungicides has a devastating impact on agricultural yields, with an average of 30% of harvests lost due to pathogenic fungi (1, 3). The four classes of fungicides most commonly used in agricultural practice include methyl benzimidazoles that target β-tubulin; strobilurins that target mitochondrial cytochrome b (complex III); succinate dehydrogenase inhibitors, which inhibit mitochondrial complex II; and demethylation inhibitors such as azoles that target 14-α-demethylase, an essential component of the ergosterol biosynthesis pathway (1). *Fusarium graminearum* is a devastating fungal pathogen that causes Fusarium Head Blight in cereal crops like wheat and barley, leading to significant yield losses and reduced grain quality. This fungal pathogen also produces harmful mycotoxins, including deoxynivalenol (DON), which contaminate crops and pose serious health risks to humans and animals (4). Azoles are most frequently deployed to combat *F. graminearum*, but often offer limited protection given the prevalence of resistance to this fungicide class (5). Other agricultural pathogens, such as the rice blast pathogen *Magnaporthe oryzae* and the generalist necrotrophic pathogen *Botrytis cinerea,* are also major threats to agricultural sustainability due to their virulence in damaging a broad range of crops and their resistance to established treatment options (6). As a consequence, there is an urgent need to develop novel strategies with broad-spectrum activity against fungal phytopathogens.

Natural products are a rich source of bioactive compounds, given that they have evolved over millions of years to interact with biological systems, often with high specificity and potency (7). Their chemical diversity makes them valuable for developing new biologically active agents, including fungicides. β-carbolines are a class of naturally occurring alkaloids produced by plants, fungi, and marine organisms (8). This class of compounds has a range of documented modes of action, including but not limited to targeting DNA topoisomerases (9), inhibiting monoamine oxidases (10), directly binding DNA (11), and inhibiting diverse cellular kinases (12). Previously, we reported inhibition of the conserved fungal dual-specificity tyrosine-regulated kinase (DYRK) Yak1 by compounds sharing a β-carboline scaffold (13). Specifically, in the opportunistic human fungal pathogen *Candida albicans*, inhibition of Yak1 by 1-acetyl β-carboline (1-ABC) or 1-ethoxycarbonyl-β-carboline (1-ECBC) prevented the yeast-to-filament transition under physiological conditions, a developmental transition important for virulence. More recently, we found that 1-carboxylic acid β-carboline (1-CABC) is effective in limiting *C. albicans* filamentation and virulence in a mouse model of dermatomycosis, similar to a *yak1* homozygous deletion mutant (14). However, given that Yak1 is dispensable for virulence in systemic and oral models of candidiasis, the therapeutic potential of β-carbolines in the treatment of *Candida-*driven disease in humans appears limited (15). Other work has explored the bioactivity of 1-ECBC in enhancing azole-efficacy *in vitro* against *Aspergillus fumigatus*, an opportunistic pulmonary fungal pathogen in humans (16), but the therapeutic potential of β-carbolines in treating aspergillosis and perhaps other pathogenic molds has yet to be explored in animal models.

Plant species are among the most prominent natural producers of β-carbolines, including those with known antifungal properties (17). Given that fungi are the most significant and devastating pathogens of plants, it is reasonable to speculate that plant species might produce β-carbolines in part as a defense against fungal infections. Thus, an investigation of whether β-carbolines could be used in agricultural settings to help control phytopathogenic fungi is warranted. Here, we report on the bioactivities of a panel of commercially available β-carbolines against the cereal pathogen *F. graminearum*. We find that several β-carbolines, including 1-CABC, inhibit *F. graminearum* conidial germination *in vitro*. Evaluating an array of common agricultural fungicides, we observe that 1-CABC is synergistic with pyraclostrobin, a strobilurin-class fungicide, in inhibiting *F. graminearum* growth, at least in large part by elevated oxidative pressure caused by the accumulation of reactive oxygen species (ROS). We also showcase that 1-CABC limits pathogen virulence, either as a single agent or in conjunction with pyraclostrobin, in models of infection for *F. graminearum,* as well as the fungal phytopathogens *B. cinerea* and *M. oryzae*. Finally, we evaluated the effects of 1-CABC on several other fungi, finding broad-spectrum bioactivity that appears to involve distinct mechanisms of action in different species. Collectively, our data provide a strong foundation to support further development of β-carbolines for the control of agricultural fungal pathogens.

## RESULTS

### 1-CABC is a priority β-carboline with fungicidal activity against *F. graminearum*

**Fig 1:**
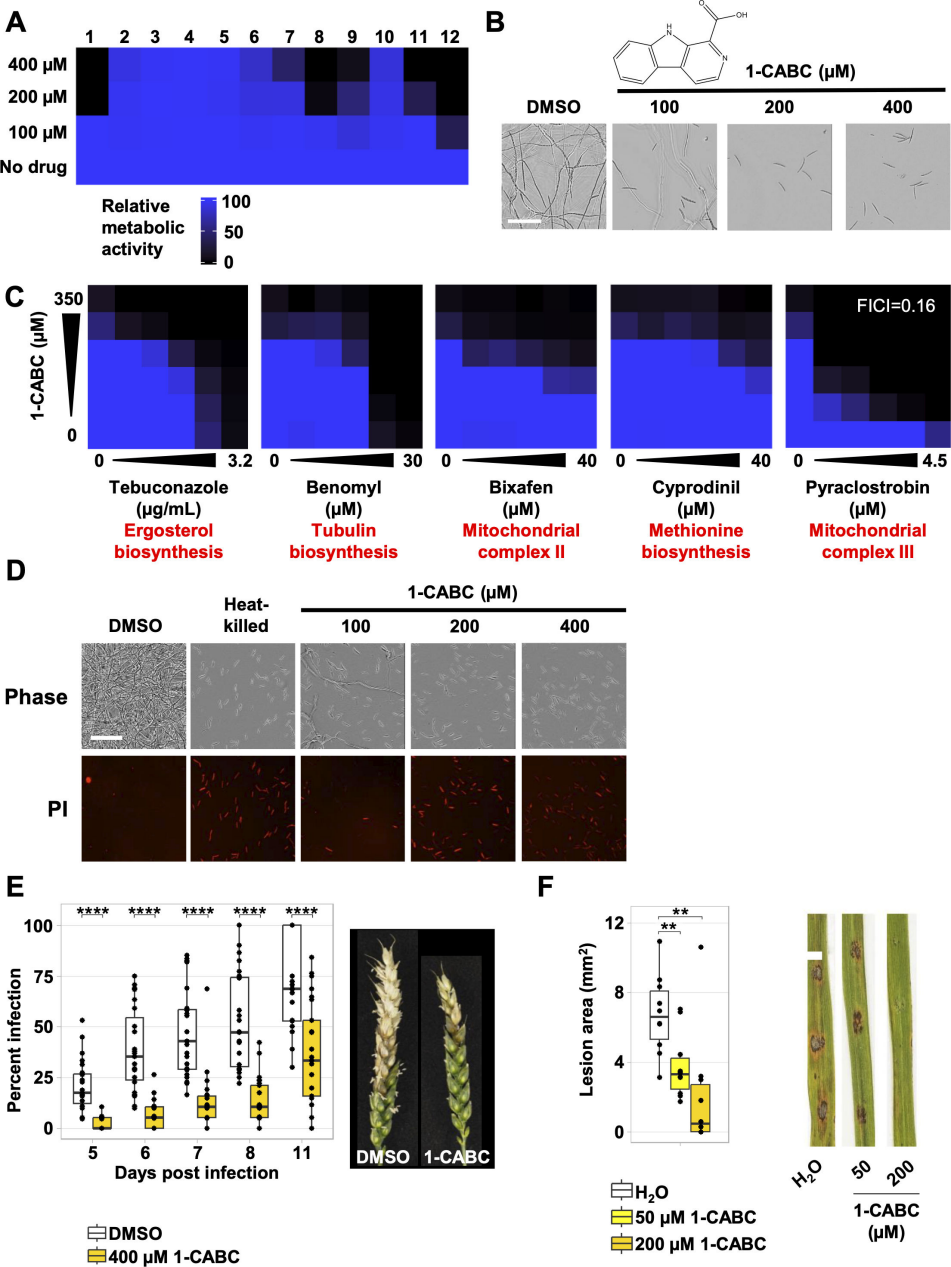
The β-carboline 1-CABC has antifungal activity against plant-pathogenic fungi. (**A**) Heatmaps denoting relative metabolic activity for *F. graminearum* in the presence of twelve distinct β-carbolines, showcasing twofold dilutions from a maximum concentration of 400 µM (2% dimethyl sulfoxide [DMSO]). Resazurin was added after 24 hours of incubation with compounds, and relative metabolic activity was quantified after another 24 hours (total incubation time = 48 hours). Color bar appears below heatmap. See Fig. S1 for names and structures of screened compounds. (**B**) Representative phase microscopy images of the effects of 1-CABC treatment on conidial germination in *F. graminearum* after a 24-hour incubation. Scale bar = 100 µm. (**C**) Checkerboard assays between 1-CABC and representative conventional fungicides reveal a synergistic interaction between 1-CABC and pyraclostrobin. Heatmaps denote the relative metabolic activity of *F. graminearum* in the presence of the indicated compound combinations (see Fig. 1A for color bar). For pyraclostrobin, the FICI_80_ is provided in the top-right corner of the heat map to indicate a synergistic interaction. A brief description of the fungicide target is provided in red beneath the fungicide name. (**D**) 1-CABC displays fungicidal activity as a single agent, as monitored by propidium iodide (PI) uptake at 18 hours. Incucyte images were taken of *F. graminearum* conidia in the presence of the indicated compound treatments and 1 µg/mL PI using both the phase and red channel every hour for >30 hours. Heat-killed conidia (95°C for 10 minutes) were used as positive controls for PI uptake. Scale bar = 200 µm. (**E**) As a single agent, 1-CABC reduces *F. graminearum* virulence following infection of mature wheat heads. Mature wheat heads were dipped in a conidial suspension (5 × 10^4^ conidia/mL) containing either 2% DMSO or 400 µM 1-CABC. The percent infection was scored over the course of 11 days by counting the number of diseased kernels relative to total kernels on each wheat head in each treatment group, giving a percent infection for each wheat head. In the box plot shown in E, each dot represents a distinct wheat head with its infection score. Statistical significance was assessed by a two-tailed unpaired t test adjusted for multiple comparisons using a Benjamini-Hochberg correction (**** =*P* < 0.0001). Representative images of wheat heads at 11 days post-infection are shown beside the graph. (**F**) 1-CABC limits *M. oryzae* infection using a detached rice leaf model of infection. A conidial suspension (15 μL; 5 × 10^4^ conidia/mL) supplemented with 1-CABC was inoculated onto detached rice leaves, with lesion development quantified 5 days post-infection via ImageJ. Significance was assessed based on a two-tailed unpaired t test adjusted using a Bonferroni multiple test correction (** =*P* < 0.01; *** =*P* < 0.001). Representative images of rice leaves at 5 days post-infection are shown below the graph.

As an initial step to determine whether β-carbolines could prove useful in agricultural settings to help control infections caused by fungi such as *F. graminearum,* we performed a small-scale *in vitro* screen of 12 commercially available β-carbolines. *F. graminearum* conidia were incubated in RPMI-1640 medium in 96-well plates with a titration of individual compounds starting at 400 μM. After 24 hours of incubation, each well was supplemented with resazurin and allowed to incubate for another 24 hours before assessing fungal metabolic activity in the presence of each compound through measurement of fluorescence (Fig. 1A; Fig. S1A). Several β-carbolines showed strong inhibition of *F. graminearum* conidial germination and metabolic activity, with harmine (9), harmane (11), norharmane (12), and 1-CABC (1) being most effective at limiting germination and reducing fungal viability (Fig. 1A and B). We prioritized 1-CABC for further investigation, given that we had previously reported antifungal efficacy for this β-carboline in a mouse model of *C. albicans* dermatitis (14).

To determine whether the bioactivity observed with 1-CABC was specific, rather than a nonspecific consequence of high concentrations of a weak acid, we first tested equimolar doses of a simple carboxylic acid, acetic acid, against *F. graminearum* and found it did not show any antifungal activity (Fig. S1B). Next, to evaluate the antifungal properties of 1-CABC and determine whether it could enhance the activity of conventional fungicides, we conducted checkerboard assays using a titration of 1-CABC alongside titrations of a representative triazole (tebuconazole), strobilurin (pyraclostrobin), succinate-dehydrogenase inhibitor (bixafen), anilinopyrimidine (cyprodinil), and a benzimidazole (benomyl). 1-CABC modestly potentiated most of the fungicides tested. In contrast, we found a synergistic interaction between 1-CABC and pyraclostrobin as defined by a fractional inhibitory concentration index (FICI) of <0.5 based on fluorescence readings after incubation with resazurin (Fig. 1C), as well as microscopy images obtained with the Incucyte Live-Cell Analysis System (Fig. S1C).

In *C. albicans,* 1-CABC blocks the yeast-to-filament transition without resulting in cell death (14). Given that our dose-response assays did not distinguish fungistatic activity versus fungicidal activity, we evaluated whether 1-CABC killed *F. graminearum*, either as a single agent or in combination with pyraclostrobin, by incubating conidia with compound treatments alongside propidium iodide, a reagent that specifically stains dead cells. We found that 1-CABC led to propidium iodide uptake in conidia after 18–24 hours at concentrations that reduced metabolic activity both alone (Fig. 1D) and in combination with pyraclostrobin (Fig. S1D). These data suggest that 1-CABC exhibits fungicidal activity after prolonged exposure, as opposed to simply blocking germination.

Finally, given its promising *in vitro* activities, we tested the efficacy of 1-CABC in protecting mature wheat heads against *F. graminearum* infection. Wheat heads were dipped in an *F. graminearum* inoculum that was supplemented with a high concentration of 1-CABC (400 μM) or an equivalent volume of DMSO solvent, and disease progression was monitored for 11 days. Encouragingly, a single 1-CABC treatment provided significant protection against *F. graminearum* disease, especially over the first week post-infection (Fig. 1E). We next probed the single-agent activity of 1-CABC against a distinct cereal pathogen, *M. oryzae*, using a detached rice leaf model of infection. Conidial suspensions of *M. oryzae* were prepared in the absence or presence of 1-CABC, a droplet of the prepared inoculum was placed on the adaxial surface of a cut rice leaf, and the inoculated leaves were incubated at 25°C. Five days post-inoculation, we observed significant compound activity with an almost complete block in *M. oryzae* disease lesion development at 200 μM (Fig. 1F). Collectively, these data highlight that β-carbolines are bioactive against phytopathogenic fungi, with 1-CABC showing *in vitro* and *in planta* antifungal activity as a single agent against representative organisms from two distinct phytopathogenic genera.

### 1-CABC enhances pyraclostrobin activity against *F. graminearum* independent of Yak1

**Fig 2:**
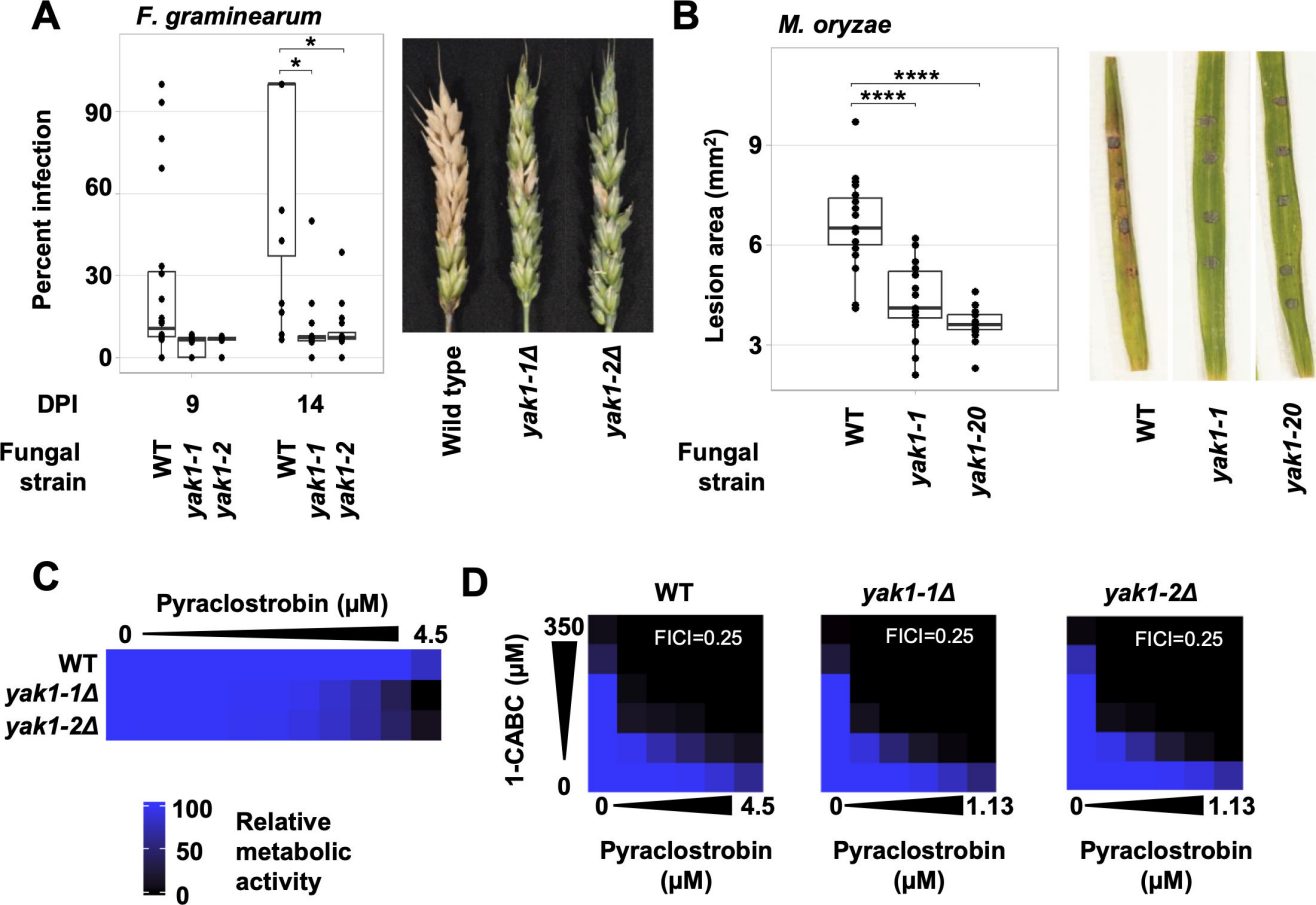
1-CABC enhances pyraclostrobin efficacy against *F. graminearum* independent of Yak1. (**A**) *YAK1* is required for full virulence in *F. graminearum*. Mature wheat head infection assays were conducted and scored as described in Fig. 1E (*=*P* < 0.05). A representative image of wheat heads infected by WT or *yak1* deletion mutants at the final timepoint is shown to the right of the quantification. (**B**) *YAK1* is required for full virulence in *M. oryzae*. Conidial suspensions from wild-type Guy11 and *yak1*Δ T1 and T20 strains were used to inoculate 14-day-old seedlings of the CO-39 rice line, and the lesion area was quantitated as described in Fig. 1F (**=*P* < 0.01). A representative image of each treatment at day 5 post-infection is shown to the right of the quantification. (**C**) Deletion of *YAK1* confers hypersensitivity to pyraclostrobin in *F. graminearum*. Two independent *yak1Δ* mutants (*yak1-1Δ* and *yak1-2Δ*) were tested for sensitivity to pyraclostrobin alongside a marker-matched wild-type control. Dose-response assay and heatmap quantitation of relative fungal metabolic activity were performed as described for Fig. 1A (color bar appears below heatmap). (**D**) Deletion of *YAK1* in *F. graminearum* does not alter the potentiation activity of 1-CABC and pyraclostrobin. Checkerboard assays and FICI calculations were performed as in Fig. 1C (see Fig. 2C for color bar).

Previously, we determined that the β-carbolines 1-ECBC and harmine inhibit Yak1, the DYRK family kinase expressed in *C. albicans,* to block the yeast-to-filament transition in this fungus (13). Yak1 has been previously characterized as dispensable for viability but required for virulence in many plant-pathogenic fungi, including *F. graminearum* strain PH-1 (18) and *M. oryzae* strain KJ201 (19), suggesting that its inhibition could be exploited for antifungal development. We confirmed that deletion of *YAK1* reduced virulence in both *F. graminearum* strain Gz3639 (Fig. 2A; *yak1-1Δ* and *yak1-2Δ*) and *M. oryzae* strain Guy11 (Fig. 2B; *yak1-1Δ* and *yak1-20Δ*), supporting Yak1 as a factor modulating, either directly or indirectly, the activity of 1-CABC against these strains.

In *F. graminearum*, deletion of *YAK1* results in hypersensitivity to oxidative stress (18). Given that strobilurin fungicides can induce oxidative stress through complex III inhibition (20, 21), and that we had observed synergy between 1-CABC and pyraclostrobin (Fig. 1C), we sought to explore whether deletion of *YAK1* had any impact on the bioactivity of 1-CABC. We used our *F. graminearum yak1Δ* mutants to evaluate whether 1-CABC treatment phenocopied the genetic deletion of this kinase. We found that *yak1Δ* mutants were hypersensitive to pyraclostrobin relative to a marker-matched wild-type control (Fig. 2C). However, deletion of *YAK1* resulted in no change in sensitivity to 1-CABC as a single agent, and the combination of 1-CABC and pyraclostrobin remained synergistic (FICI <0.5) in all strains (Fig. 2D). Overall, observations thus far suggest that while 1-CABC may inhibit Yak1 to impair virulence, the ability of 1-CABC to enhance pyraclostrobin efficacy is not mediated through inhibition of Yak1.

### 1-CABC and pyraclostrobin synergy is driven by intracellular ROS accumulation and mitochondrial damage

**Fig 3:**
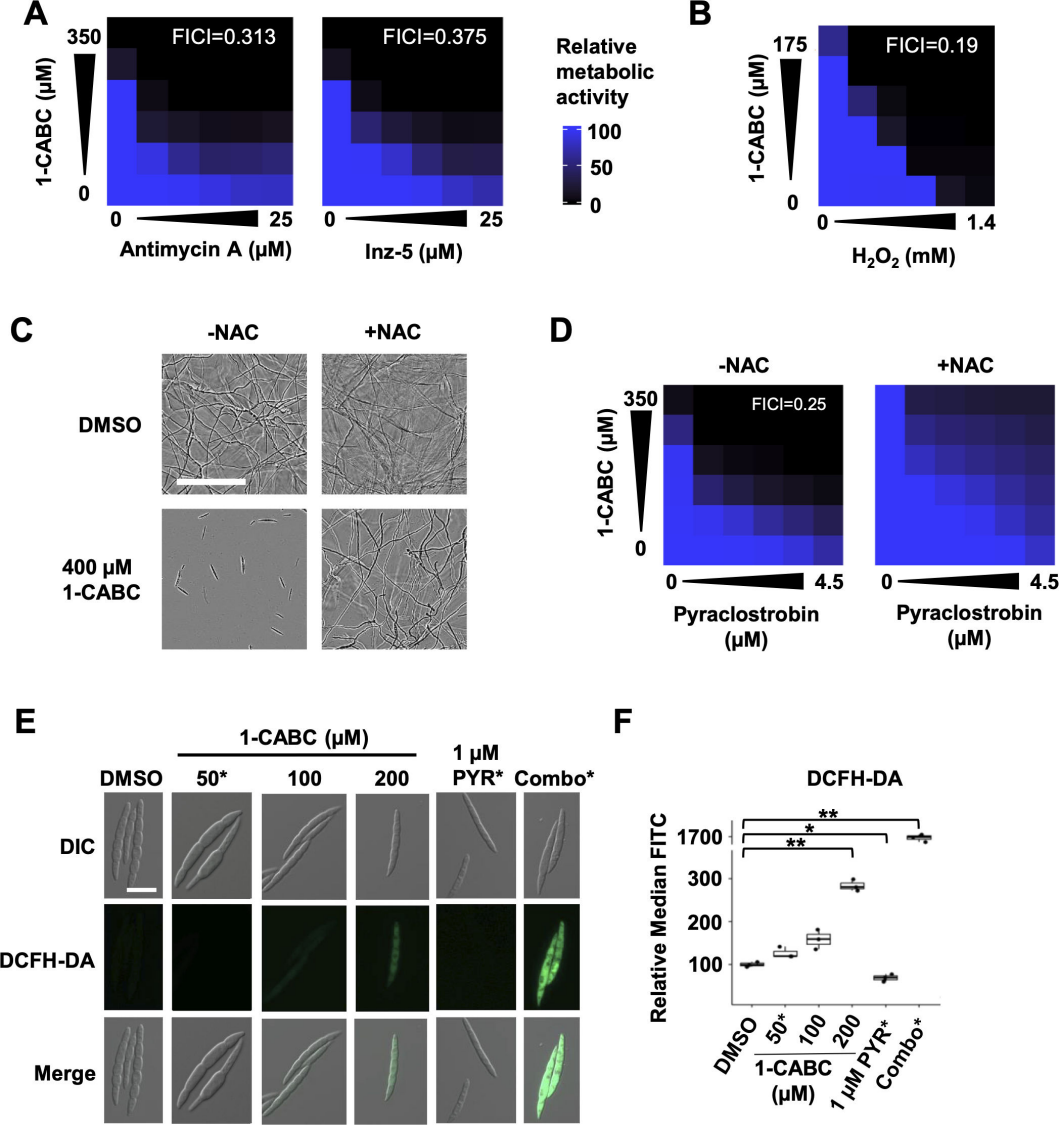
1-CABC bioactivity and synergy with mitochondrial complex III inhibitors are associated with ROS induction and mitochondrial damage. (**A**) 1-CABC synergizes with structurally diverse mitochondrial complex III inhibitors, Antimycin A and Inz-5. Checkerboard assays were performed and visualized as described in Fig. 1C. (**B**) 1-CABC synergizes with H_2_O_2_ against *F. graminearum*. Checkerboard assays were performed and visualized as in Fig. 1C. (**C and D**) Supplementation with NAC abolishes 1-CABC bioactivity and dampens the interaction between 1-CABC and pyraclostrobin. (**C**) Representative phase microscopy images of *F. graminearum* conidia after incubation with 400 µM 1-CABC in the presence or absence of 5 mM NAC. Images were taken after a 24-hour incubation. Scale bar = 200 µm. (**D**) Checkerboard assays and FICI calculations were performed and visualized as in Fig. 1C. (**E and F**) 1-CABC and pyraclostrobin lead to ROS accumulation in *F. graminearum* as measured with the fluorescent ROS probe DCFH-DA. (**E**) Representative fluorescence microscopy of *F. graminearum* conidia after incubation with the indicated compounds and staining with DCFH-DA (left). Scale bar = 20 µm. (**F**) Quantification of intracellular ROS after DCFH-DA staining by flow cytometry. The median FITC-A values across 10,000 events for three distinct experiments were averaged and normalized as a percentage to the DMSO control to evaluate statistically significant deviations from DMSO fluorescence with a paired t-test (* =*P* < 0.05; ** =*P* < 0.01).

Strobilurins inhibit cellular respiration by inhibiting mitochondrial complex III, resulting in impaired ATP generation, mitochondrial dysfunction, and the generation of ROS, leading to oxidative stress (20, 21). To assess whether the synergy observed between 1-CABC and pyraclostrobin against *F. graminearum* was specific to strobilurins or was conserved with other mitochondrial inhibitors, we tested 1-CABC in checkerboard assays with two structurally distinct complex III inhibitors, Inz-5 and Antimycin A. Upon incubation of *F. graminearum* conidia with compound combinations, we found that synergy between 1-CABC and the alternate mitochondrial inhibitors was conserved, with FICI values < 0.5 (Fig. 3A).

Next, we explored whether the synergistic interaction between 1-CABC and complex III inhibitors was due to enhanced impairment of mitochondrial function. To do so, we treated *F. graminearum* with compound for 1 hour and subsequently stained conidia with MitoTracker Red, a dye that selectively and irreversibly stains mitochondria in a membrane potential-dependent manner. We found that 1-CABC reduced mitochondrial membrane potential in a dose-dependent manner as quantified with flow cytometry (Fig. S2A and B; gating strategy in Fig. S2C) and visualized by fluorescence microscopy (Fig. S2D). Given that prolonged exposure to 1-CABC has fungicidal activity against *F. graminearum* (Fig. S1D and E), we confirmed that conidia were not killed in the 1-hour timeframe used to examine mitochondrial membrane potential by propidium iodide staining, using 0.5% SDS as a positive control for cell death (Fig. S2E), verifying changes observed in the flow cytometric analysis were due to changes in viable conidia. However, we found that while 1-CABC and pyraclostrobin lowered mitochondrial membrane potential as single agents, the effects of the combination were statistically indistinguishable from the single compound treatments (Fig. S2F and G), suggesting that the synergistic effects of 1-CABC and pyraclostrobin are unlikely to be due to an effect on mitochondrial membrane potential.

Given that oxidative stress due to electron leakage is a potential consequence of severe mitochondrial depolarization (22, 23), we explored whether 1-CABC bioactivity was synergistic with oxidative stress in general. Indeed, a checkerboard assay performed with 1-CABC and hydrogen peroxide (H_2_O_2_) confirmed that our compound synergized with this general oxidative stressor in inhibiting *F. graminearum* growth (Fig. 3B). To probe whether observed synergies were ROS-dependent, we investigated the 1-CABC-pyraclostrobin interaction in the presence and absence of N-acetyl L-cysteine (NAC) or L-glutathione, two distinct ROS scavengers (24). Supplementing medium with NAC or L-glutathione abolished the single agent activity of 1-CABC and dampened the interaction between 1-CABC and pyraclostrobin (Fig. 3C and D; Fig. S3A and B), supporting that the single-agent and pyraclostrobin-potentiating activity of 1-CABC are driven by ROS accumulation. Since both NAC and L-glutathione are thiol-containing compounds, we confirmed that 1-CABC did not chemically react with the thiol moiety in NAC using Ellman’s reagent (5,5′-dithiobis(2-nitrobenzoic acid)), which can be used to monitor the concentration of free thiols in solution (25). In contrast to the established thiol-reactive compound N-ethylmaleimide (NEM), 1-CABC did not diminish the colored product generated by incubation of Ellman’s reagent with NAC (Fig. S3C), confirming that 1-CABC does not generically adduct thiol-containing compounds. Thus, the effects of NAC and glutathione on 1-CABC bioactivity are driven by their antioxidant properties in cells, rather than direct chemical inactivation of 1-CABC.

Finally, we investigated the role of ROS accumulation on the synergistic interaction between 1-CABC and pyraclostrobin using 2′,7′-dichlorodihydrofluorescein diacetate (DCFH-DA), a fluorescent probe that reports relative intracellular ROS levels. Increasing concentrations of 1-CABC led to a dose-dependent increase in ROS accumulation as observed by microscopy and as quantified by flow cytometry (Fig. 3E and F; Fig. S3D). Furthermore, a combination of a subinhibitory concentration of 1-CABC (50 µM) and a subinhibitory concentration of pyraclostrobin (1 µM) led to a significant increase in intracellular ROS accumulation, indicating that 1-CABC and pyraclostrobin synergistically increase oxidative stress in *F. graminearum* conidia. Importantly, a ROS-insensitive analog of DCFH-DA, 2′,7′-dichlorofluorescein diacetate (DCF-DA), did not have significantly altered fluorescence in the presence of our compound treatments (Fig. S3E), highlighting that differences observed with DCFH-DA were due to genuine changes in intracellular ROS and not due to differential dye accumulation. Additionally, we confirmed that 1-CABC treatment alone did not have any intrinsic fluorescence relative to an unstained DMSO control (Fig. S3F), further confirming that the differences observed in DCFH-DA fluorescence intensity are due to ROS accumulation. Overall, these observations support a model where synergistic interaction between pyraclostrobin and 1-CABC is driven primarily by ROS accumulation.

### 1-CABC and harmane inhibit *F. graminearum* growth through distinct mechanisms

**Fig 4:**
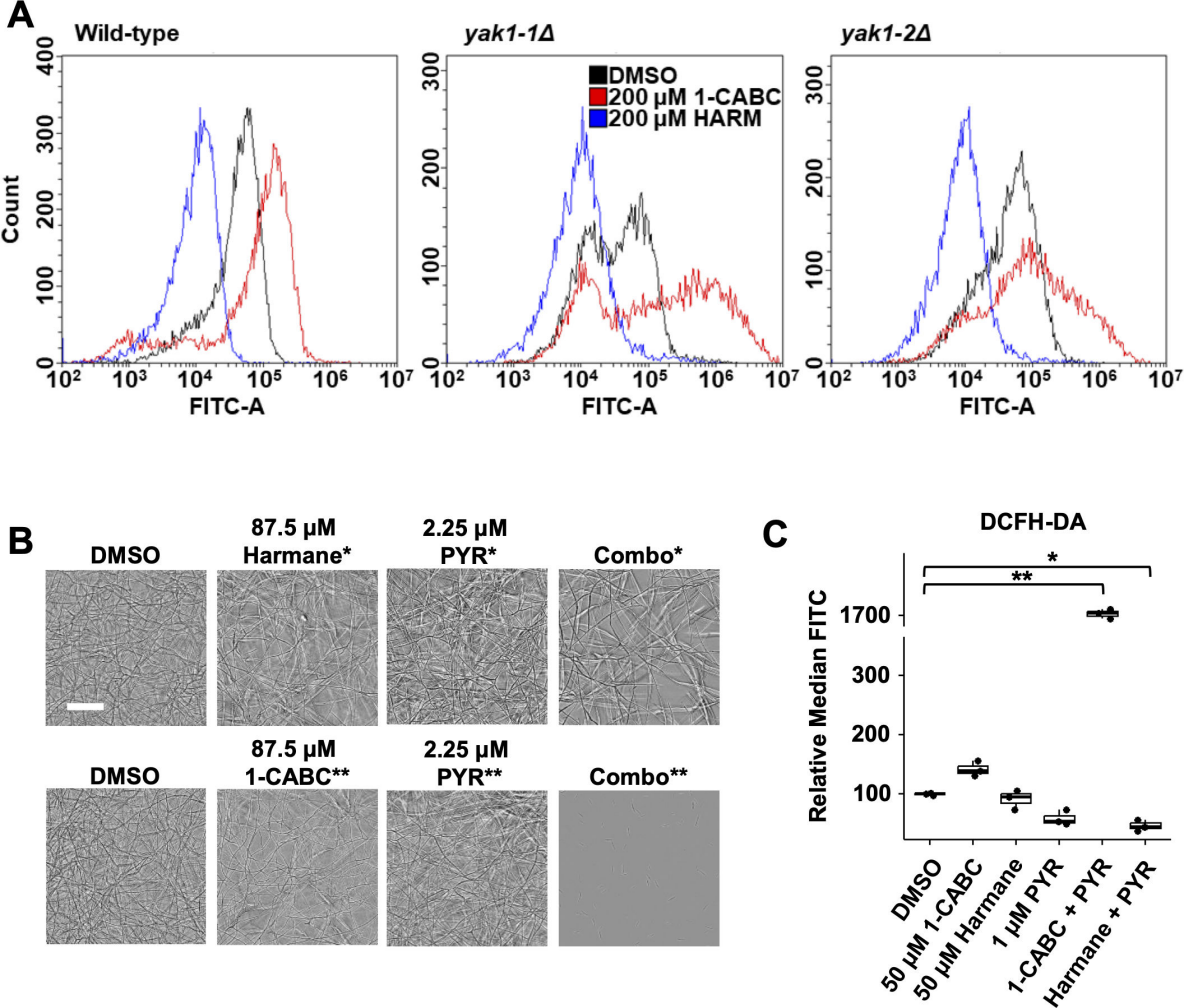
1-CABC and harmane inhibit *F. graminearum* conidial germination through distinct mechanisms. (**A**) 1-CABC enhances intracellular ROS accumulation independently of *YAK1*. Strains were exposed to the indicated compound treatments, and samples were subjected to flow cytometry on the green fluorescence channel (FITC-A) after staining with DCFH-DA. Harmane (HARM), a distinct beta-carboline with established antioxidant properties, diminishes intracellular ROS accumulation, whereas 1-CABC enhances ROS production in both wild-type and *yak1* mutants. (**B**) Harmane does not enhance the activity of pyraclostrobin. Phase images after a 48-hour incubation were used to highlight the lack of synergy when compared to 1-CABC. Scale bar = 200 µm. (**C**) 1-CABC and harmane have opposing effects on intracellular ROS accumulation in *F. graminearum* when used in combination with pyraclostrobin. Flow cytometry was conducted as described for Fig. 3F. The median FITC-A values across 10,000 events for three distinct experiments were averaged and normalized as a percentage of the DMSO control to evaluate statistically significant deviations from fluorescence of the control cell population with a paired t-test (* =*P* < 0.05; ** =*P* < 0.01).

Our observation that 1-CABC induces oxidative stress was surprising, given that other well-studied β-carbolines, including harmane and harmine, are documented to have antioxidant properties in both fungal and human cells (26, 27). To further explore this differential activity, we examined the effects of 1-CABC or harmane on ROS levels in *F. graminearum* conidia, once again using DCFH-DA. We tested wild-type and *yak1Δ* conidia to both evaluate the effects of 1-CABC on intracellular ROS accumulation and to determine whether the effects were *YAK1*-dependent. Treatment with 200 µM harmane led to a decrease in intracellular ROS accumulation in *F. graminearum*, while the same concentration of 1-CABC resulted in an increase in ROS accumulation (Fig. 4A). Furthermore, *F. graminearum yak1Δ* mutants still accumulated ROS in the presence of 1-CABC, albeit with more heterogeneity in fluorescent signal, highlighting that ROS accumulation in the presence of 1-CABC is not abolished in the absence of *YAK1*. Given this altered ROS distribution, we confirmed that *yak1Δ* mutants were not killed in the presence of the indicated treatments using propidium iodide followed by flow cytometry (Fig. S3G).

Given that harmane reduced intracellular ROS in *F. graminearum*, we hypothesized that harmane would not potentiate pyraclostrobin in limiting *F. graminearum* growth. Indeed, a combination of harmane and pyraclostrobin did not inhibit conidial germination as visualized by microscopy, unlike what was observed with an equal concentration of 1-CABC (Fig. 4B). We also found that harmane and pyraclostrobin in combination resulted in a decrease in ROS accumulation than either compound in isolation (Fig. 4C; Fig. S3H). These data suggest that while both 1-CABC and harmane are active against *F. graminearum*, the nature of their antifungal activity is distinct and is not changed with the deletion of *YAK1*.

### 1-CABC displays efficacy in multiple models of plant infection

**Fig 5:**
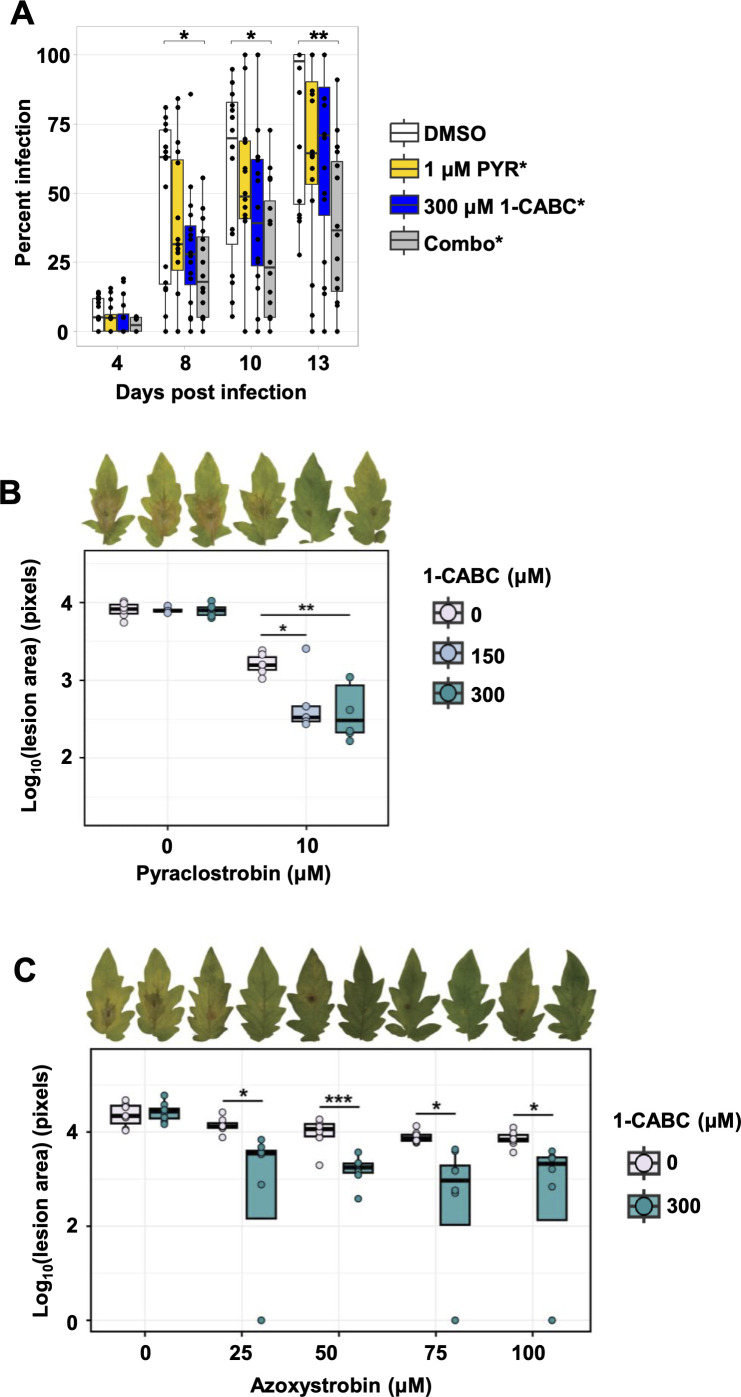
1-CABC has *in planta* efficacy in several models of plant fungal infection. (**A**) A combination of 1-CABC and pyraclostrobin (PYR) exhibits greater efficacy relative to individual treatments in a *F. graminearum* mature wheat head infection model. Infections were conducted as in Fig. 1E (* =*P* < 0.05, ** =*P* < 0.01). (**B**) 1-CABC displays no single-agent efficacy against *Botrytis cinerea* but enhances pyraclostrobin efficacy to limit virulence in a detached tomato leaf assay. Lesion size was quantified using Colour-analyzer; representative tomato leaf images are placed above each treatment. Significance was assessed within treatment groups by ANOVA followed by a post hoc Dunnett’s test (* =*P* < 0.05; ** =*P* < 0.01). (**C**) 1-CABC improves azoxystrobin efficacy against *B. cinerea* in a detached tomato leaf assay. Detached leaf assays were performed and analyzed as in Fig. 5B, and significance was assessed within treatment groups by ANOVA followed by a t-test (* =*P* < 0.05; *** =*P* < 0.001).

While antifungal properties of β-carbolines have been reported (28; 29; 30), investigations into the agricultural relevance of these compounds and, in particular, 1-CABC have been limited. Thus, we investigated whether the synergy observed between 1-CABC and pyraclostrobin *in vitro* could be translated to infection of mature wheat heads by *F. graminearum*. Wheat heads were dipped in an *F. graminearum* inoculum that was supplemented with vehicle, 1-CABC (300 μM), pyraclostrobin (1 μM), or the combination, and disease progression was monitored for 13 days. As predicted based on the *in vitro* findings, a combination of 1-CABC and pyraclostrobin reduced virulence relative to individual treatments up to 5 days post-infection (Fig. 5A). We saw similar, though less pronounced, results when 200 μM 1-CABC was used for single-agent and combination tests (Fig. S4). Next, we explored whether 1-CABC could improve pyraclostrobin efficacy against a distinct plant pathogenic fungus, the generalist *B. cinerea*. Fungal conidial suspensions containing compounds or vehicle control were dropped on the midvein of detached tomato leaves, and the samples were left under ambient light at room temperature in a humidified environment for 4 days to allow for lesion development. 1-CABC displayed no activity against *B. cinerea* as a single agent, as measured through quantification of the leaf lesion size, but was able to potentiate pyraclostrobin and decrease lesion development when applied in combination (Fig. 5B). We also found that azoxystrobin, a structurally-distinct strobilurin with poor single-agent activity against *B. cinerea*, had its activity improved in combination with 1-CABC (Fig. 5C), highlighting that this β-carboline can sensitize fungi to ineffective strobilurins. Collectively, these data demonstrate the potential of 1-CABC to improve conventional fungicide efficacy *in planta* against two distinct fungal phytopathogens.

### 1-CABC has activity against diverse fungi through distinct modes of action

**Fig 6:**
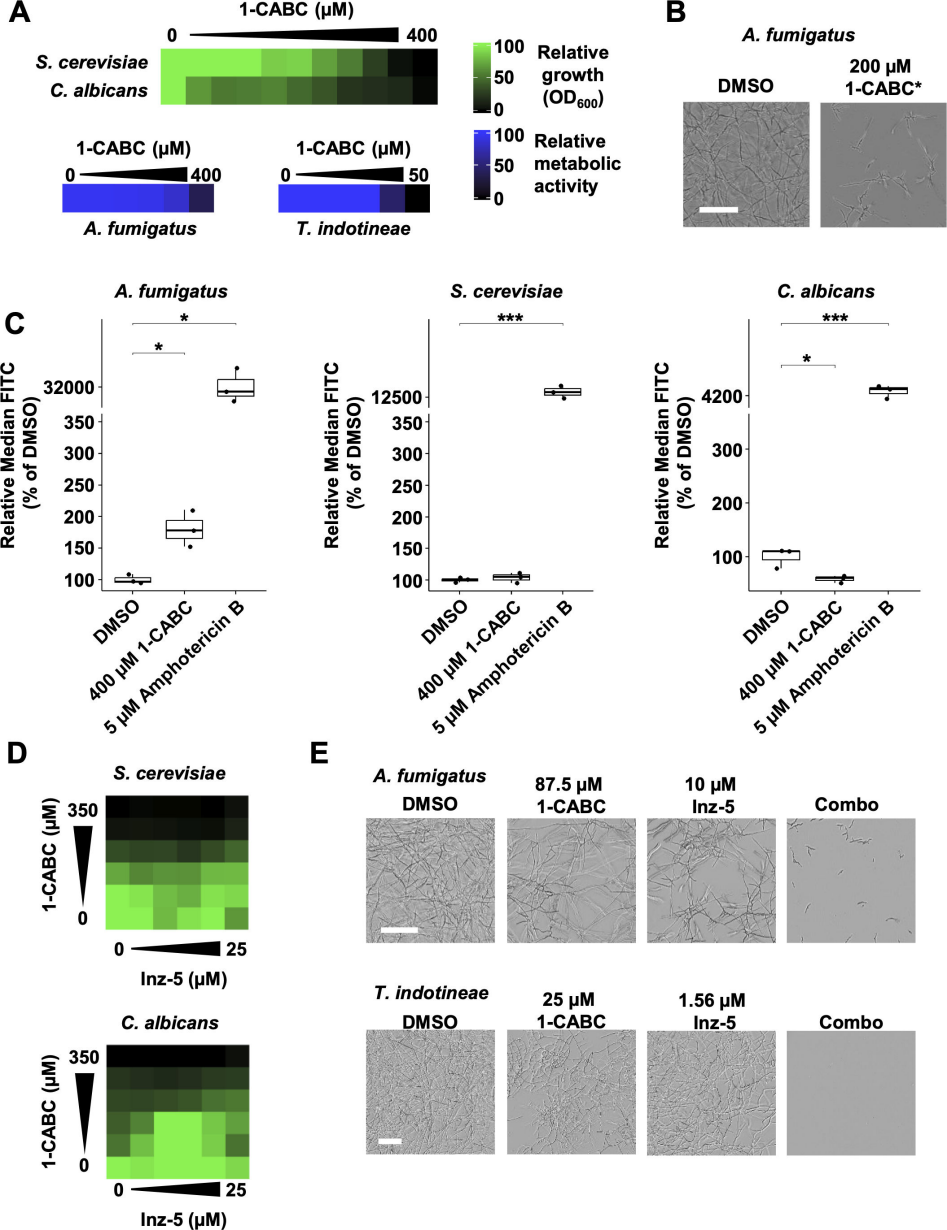
1-CABC exerts broad-spectrum single-agent antifungal activity but only potentiates mitochondrial complex III inhibitors in select molds. (**A**) Dose-response assays for 1-CABC against diverse fungal species after a 48-hour incubation. For the yeasts, *C. albicans* and *S. cerevisiae*, growth was measured via OD_600_, normalized to a no-drug control, and visualized as a green-to-black heat map denoting fungal growth (see color bar). For *A. fumigatus* and *T. indotiniae*, the metabolic activity was assessed as described in Fig. 1A, except for *T. indotiniae,* metabolic activity was assessed after a 96-hour incubation. (**B**) 1-CABC is bioactive against *A. fumigatus* at earlier incubation timepoints (24 hours). Representative phase microscopy images. Scale bar = 200 µm. (**C**) DCFH-DA staining after 1-CABC treatment in *A. fumigatus*, *S. cerevisiae*, and *C. albicans*. All species were treated with 400 μM 1-CABC and stained with DCFH-DA in a similar manner to *F. graminearum* in Fig. 3E. DMSO and 5 μM amphotericin B were included as negative and positive controls for ROS accumulation, respectively. Quantification of relative intracellular ROS accumulation was monitored by DCFH-DA staining and flow cytometry. The median FITC-A values across 10,000 events for three distinct experiments were averaged and normalized as a percentage to the DMSO control to evaluate statistically significant deviations from DMSO fluorescence with a paired t-test (* =*P* < 0.05; ** =*P* < 0.01). (**D**) Checkerboard assays between 1-CABC and Inz-5 in *S. cerevisiae* (top) and *C. albicans* (bottom). Growth was monitored as described in panel A. (**E**) Potentiation of Inz-5 and 1-CABC in *A. fumigatus* (top) and *T. indotineae* (bottom). Representative phase images. Scale bar = 200 µm.

The efficacy of 1-CABC against multiple plant fungal pathogens led us to explore its *in vitro* bioactivity against a broader range of fungi, including the model yeast *Saccharomyces cerevisiae* and a panel of opportunistic pathogens of humans: *C. albicans, A. fumigatus,* and *Trichophyton indotineae*. We found that this β-carboline was active against all tested fungi, though with varying degrees of potency (Fig. 6A). The least potent antifungal activity of 1-CABC was observed against *A. fumigatus*, with partial growth inhibition detected after 24 hours at 200 µM (Fig. 6B), and a minimum inhibitory concentration required to reduce metabolic activity by 50% (MIC_50_) of 400 µM after 48 hours (Fig. 6A). In contrast*,* 1-CABC was effective at lower concentrations, with MIC_80_ values of 200 µM and 400 µM against *S. cerevisiae* and *C. albicans*, respectively. Interestingly, *T. indotineae* was the most sensitive to 1-CABC, with an MIC_80_ of 50 µM.

Given our finding that 1-CABC antifungal activity against *F. graminearum* is closely associated with ROS accumulation, we explored the extent to which ROS accumulation is associated with the activity of 1-CABC against other fungal species. We conducted flow cytometry after DCFH-DA staining for *S. cerevisiae*, *C. albicans,* and *A. fumigatus*, both in the presence and absence of 400 µM 1-CABC, with similar media conditions to those used for *F. graminearum*. We selected this single high concentration as it showed growth-inhibitory activity against all fungal species examined. Furthermore, we used amphotericin B as a positive control for ROS induction, as it results in ROS accumulation in diverse fungal species (31). Intriguingly, we found that 1-CABC resulted in significant ROS accumulation only in *A. fumigatus* and had either no effect on ROS accumulation in *S. cerevisiae* or a significant antioxidant effect in *C. albicans* (Fig. 6C, Fig. S5A and B). In contrast, amphotericin B resulted in a significant increase in ROS accumulation across all species. Given that 1-CABC results in a synergistic interaction with complex III inhibitors in *F. graminearum,* we explored whether 1-CABC was able to potentiate Inz-5 in our extended fungal panel. We found that 1-CABC had either no interaction or slight antagonism with Inz-5 when tested against *S. cerevisiae* and *C. albicans*, respectively (Fig. 6D); while in contrast, 1-CABC potentiated Inz-5 activity in *A. fumigatus* (Fig. 6E, top). While we did not conduct flow cytometric analysis with *T. indotineae,* we found that 1-CABC potentiated Inz-5 in this species (Fig. 6E, bottom), suggesting that 1-CABC induces ROS production selectively in molds. Overall, this work highlights the broad therapeutic potential of 1-CABC against diverse agricultural fungal pathogens while also emphasizing the distinct biological activities of two different β-carbolines and the unique association that a single β-carboline can have in inducing ROS across different fungal species.

## DISCUSSION

β-carbolines comprise a class of diverse alkaloids, many of which are natural products produced by plants (17). A wide range of bioactivities has been ascribed to β-carbolines, though often with differing levels of potency and specificity depending on the β-carboline tested. In our study, the priority compound 1-CABC had similar antifungal activity against *F. graminearum* when compared to well-studied β-carbolines such as harmane, but its mode of action against this organism was distinct in that it induced, rather than reduced, ROS levels. This finding aligns with previous studies that have characterized the influence of structural modifications to the β-carboline scaffold on the compound’s mechanism of action. For instance, harmine (compound 9 in our panel) has been reported as an inhibitor of both human monoamine oxidase A (MAO-A) as well as dual-specificity tyrosine phosphorylation-regulated kinase 1A (DYRK1A), though simple modifications to its structure can preserve its activity against DYRK1A while compromising its inhibition of MAO-A (32). Similarly, several β-carbolines at sufficiently high concentrations bind DNA, though the potency and nature of this interaction can vary substantially when comparing β-carbolines with minor structural differences (11). Interestingly, the divergence in ROS induction between 1-CABC and harmane—perhaps due to the zwitterionic properties of the carboxylic acid moiety of 1-CABC—is what we predict enables 1-CABC to synergize with strobilurin-class mitochondrial inhibitors, highlighting the potential of the compound combination as a fungicide treatment. 1-CABC was also efficacious as a single agent and in combination with strobilurins in controlling diseases caused by *M. oryzae* and *B. cinerea*, suggesting that 1-CABC has broad-spectrum activity against plant-pathogenic molds.

Intriguingly, 1-CABC did not universally induce ROS accumulation in fungi, despite retaining bioactivity against all species tested. Exposure to growth-inhibitory concentrations of 1-CABC resulted in ROS accumulation in the filamentous fungi *F. graminearum* and *A. fumigatus* but had no such effect on the yeasts *S. cerevisiae* and *C. albicans*. One hypothesis for this discrepancy is that 1-CABC has conserved target(s) across fungal species, but that compromising such target(s) only results in ROS accumulation in filamentous fungi. Alternatively, there may be divergence in the cellular target(s) of 1-CABC in different fungal species. One could hypothesize that β-carbolines such as 1-ABC, 1-ECBC, and 1-CABC inhibit *C. albicans* Yak1 to block the yeast-to-filament transition, whereas the kinase is sufficiently divergent in molds such that 1-CABC inhibits a different cellular target to induce ROS production and synergize with complex III mitochondrial inhibitors in these distinct fungal pathogens. While we found no genomic evidence of *F. graminearum* or other pathogenic molds having an expanded array of DYRK-family kinases relative to yeasts, it is possible that 1-CABC achieves its specific effects in molds through inhibition of multiple DYRK-family kinases, such as the related DYRK Pom1 (18). Future work will be needed to decode the full spectrum of interactions between β-carbolines like 1-CABC and DYRK-family kinases.

The *One Health Perspective* draws connections between our efforts to control both clinical and agricultural pathogens (2). Alarmingly, increasing evidence suggests that the use of similar antifungal strategies in clinical and agricultural settings (e.g., azoles) is driving cross-resistance in populations of human pathogenic fungi (33), necessitating us to develop non-overlapping strategies for dealing with clinical and agricultural pathogens. In this work, we highlighted the potential of 1-CABC in treating a range of fungal-borne plant diseases through a distinct mode of action from currently marketed fungicides. The synergistic interaction between 1-CABC and strobilurins suggests that β-carbolines could be effective adjuvants in strobilurin formulations, particularly given that these drugs are high-risk fungicides for which single-site mutations dramatically reduce their efficacy (1, 34). While future work will be needed to better understand the underlying mechanism by which 1-CABC drives ROS generation, we have nonetheless shown that this β-carboline can improve the efficacy of an established fungicide class, both *in vitro* and *in planta*. Notably, other work has proposed β-carbolines as azole potentiators for treating diseases caused by *A. fumigatus* (16), and we highlight the potential of 1-CABC with mitochondrial complex III inhibitors to treat drug-resistant dermatomycoses caused by *T. indotineae*. Thus, continued exploration of the antifungal properties of β-carbolines is required to evaluate their optimal application to combat diverse fungal pathogens.

## MATERIALS AND METHODS

### β-carboline panel

All β-carbolines stock solutions were prepared in DMSO at 20 mM and were used within a month of formulation (Table S1). All other fungicides were also prepared in DMSO at a stock concentration of 10 mM, with the exception of tebuconazole, which was prepared at a stock concentration of 5 mg/mL. All compounds were stored at −20°C.

### *Fusarium graminearum* strain information and conidial isolation

The *F. graminearum* strain DOAM 233423 (also known as Gz3639) was received from Agriculture and Agri-Food Canada, Ottawa, ON. To generate conidia, a mycelial plug of Gz3639 was transferred from a potato dextrose agar (PDA) plate to an Erlenmeyer flask containing 50 mL of carboxymethyl cellulose (CMC) medium (recipe per liter: 15 g CMC sodium salt, 1 g NH_4_NO_3_, 1 g KH_2_PO_4_, 0.5 g MgSO_4_ · 7H_2_O, 1 g yeast extract, brought up to 1 L with ddH_2_O) and grown at 28°C in the dark with 190 rpm shaking for 3–5 days. The contents of this flask were then passed through four layers of autoclaved cheesecloth or Miracloth to separate conidia from filaments. Conidia were centrifuged at 4,500 rpm for 10 minutes, and the supernatant was discarded. The conidia were then washed with 30 mL sterile ddH_2_O three times, resuspended in a final volume of 5–10 mL ddH_2_O, and counted with a hemacytometer. Conidia at a concentration greater than 1 × 10^6^ conidia/mL were stored for up to 3 weeks at 4°C. For a complete list of fungal strains used in this study, see Table S2.

### *In vitro* antifungal drug susceptibility testing in *F. graminearum*

For all *in vitro* experiments, *F. graminearum* conidia were added to 96-well plates at a starting density of 1.25 × 10^4^ conidia/mL, in a final volume of 200 μL. RPMI medium was used for all *in vitro* work in this study (recipe per liter: 10.4 g RPMI-1640, 3.5% MOPS, brought up to 1 L with ddH_2_O). Compounds were prepared at 2× the desired concentration in 100 μL RPMI and supplemented with 100 μL of RPMI containing 2.5 × 10^4^ conidia/mL. Upon the addition of conidia, 96-well plates were incubated at 28°C for 24 hours and then imaged using the Incucyte S3 Live-Cell Analysis System (Sartorius) at 20× magnification to visualize the impact of each compound on conidial germination. A total of 20 μL of RPMI supplemented with 3 mg/mL resazurin (sodium salt) was then added to each well, and plates were returned to incubate at 28°C for 24 hours. After 48 hours, plates were read using a TECAN Infinite 200 Pro (Ex. 530 nm/Em. 590 nm). All compound treatments were normalized to a no-drug control to produce a relative metabolic rate to this control. For testing the effects of NAC supplementation on compound activity, 5 mM NAC was added to RPMI, and the mixture was filter-sterilized (0.2-micron filter) before adding compound treatments and *F. graminearum* conidia. Data visualizations were produced using R.

For checkerboard assays, FICI values were calculated as described previously (35). Fractional inhibitory concentrations (FICs) were identified as concentrations of each compound (either alone or in combination) that inhibited growth by roughly 80% (MIC_80_ values). The following calculation was then performed: FICI = (MIC_80_[Compound 1 in combination]/MIC_80_[Compound 1 alone]) + (MIC_80_[Compound 2 in combination]/MIC_80_[Compound 2 alone]). If the highest concentration tested for a given compound did not lead to 80% inhibition, the MIC_80_ was estimated as twofold higher than the highest tested concentration. Instances of synergy (FICI <0.5) were highlighted in the top-right corner of the visualization for this interaction.

### *In vitro* drug susceptibility with non-*Fusarium* species

To generate *A. fumigatus* conidia, a glycerol stock of *A. fumigatus* A1163 (AfLC9065) was inoculated onto a PDA plate and incubated at 37°C for 48 hours. To harvest *A. fumigatus* conidia, a sterile cell scraper was used to transfer conidia from the PDA plate into a sterile 50 mL conical containing roughly 10 mL of filter-sterilized Tween saline solution (per liter: 0.025% [vol/vol] Tween 80, 8 g NaCl, ddH_2_O). This tube was then passed through a 40-micron filter to isolate *A. fumigatus* conidia. Purified *A. fumigatus* conidia were then counted using a hemocytometer to determine the conidial concentration. For *in vitro* drug susceptibility testing, *A. fumigatus* conidia were prepared and tested against compounds in a similar manner to *F. graminearum* (RPMI medium, starting density of 1.25 × 10^4^ conidia/mL, 3 mg/mL resazurin, 48-hour read-out); only *A. fumigatus* conidia were incubated at 37°C over the course of the experiment.

For *T. indotineae* (TiLC9502), conidia were generated by streaking from a glycerol stock onto a PDA plate and incubating at 30°C for 4–5 days. 1× PBS was added to the plate, and conidia were resuspended in the PBS using a sterile cell scraper and transferred to a sterile 50 mL conical tube. The mixture was vortexed for 1 minute and then allowed to rest for 5–10 minutes to allow large clumps and hyphae to settle. Then, 10 µL of the supernatant was taken, and conidia were counted using a hemocytometer. Drug susceptibility assays were set up in 96-well plates in a manner similar to *A. fumigatus* (200 µL final inoculum in fungal RPMI, only with a final conidial concentration of 4 × 10^3^ conidia/mL) and incubated at 35°C for 72 hours. After 72 hours of incubation, plates were imaged using the Incucyte S3 Live-Cell Analysis System (Sartorius) at 4× magnification to visualize the effects of compound treatment on conidial germination. After imaging, 50 µL of a freshly made alamarBlue solution (alamarBlue Cell Viability Reagent [Thermo Scientific DAL1100] mixed 1:3 with RPMI and sterilized through a 0.2-micron filter) was added to each well. Plates were returned to 35°C for another 24 hours, and then metabolic activity was quantified using a TECAN Infinite 200 Pro to assess the (Ex. 530 nm/Em. 590 nm), with experiments being normalized in a similar fashion to what was done for *A. fumigatus*.

For *C. albicans* CaSS1 (CaLC6106) and *S. cerevisiae* BY4741 (ScLC151), strains were streaked from glycerol onto YPD agar plates and incubated for 48 hours at 30°C. A single colony was then inoculated into 10 mL YPD and incubated overnight at 30°C, 220 rpm. Overnight yeast cultures were diluted to a starting OD_600_ of 0.005 in RPMI supplemented with 2% glucose and several amino acids (20 mg/L histidine-HCl, 100 mg/L L-leucine, 50 mg/L L-arginine-HCl, 20 mg/L L-methionine, and 20 mg/L uracil) to address auxotrophies of the *C. albicans* and *S. cerevisiae* strains used (hereafter RPMI +AAs). A total of 100 μL of this 2× inoculum was then added to 96-well plates with 2× of the intended drug concentrations in 100 μL, as described above for *F. graminearum*, for a total volume of 200 μL at a starting OD_600_ of 0.0025. Plates were covered in aluminum foil and incubated at 30°C for 48 hours, taking OD_600_ readings every 24 hours.

### Quantification of ROS accumulation and mitochondrial membrane potential in *F. graminearum*

To quantify intracellular ROS accumulation in the presence of β-carbolines and/or pyraclostrobin, Gz3639 conidia were inoculated in RPMI at a concentration of 2.5 × 10^5^ conidia/mL, separated into 1 mL aliquots, and incubated at 28°C in the dark with 190 rpm for 1 hour. Compound treatments (or an equivalent concentration of DMSO) were then added to individual 1 mL aliquots, and the conidia were returned to the incubator for 1 hour. After 1-hour incubation with compound treatments, 2.5 μg/mL DCFH-DA (Sigma 35845; stock concentration at 5 mg/mL) was added to each tube, and cultures were returned to the incubator for 30 minutes. After 30-minute incubation with the stain, 250 μL of each sample was immediately loaded onto a flow cytometer. In all, 10,000 conidia were assessed based on our gating strategy (Fig. S2A) for green fluorescence intensity (FITC-A). The same strategy was used for staining conidia with the ROS-insensitive dye DCF-DA (Sigma 21884, stock concentration at 5 mg/mL). For assessing mitochondrial membrane potential in *F. graminearum*, the same protocol was used; only conidia were stained after compound incubation with 250 nM MitoTracker Red CMXRos (Thermo Scientific M7512, stock concentration at 0.1 mM) for 30 minutes before immediately loading onto a flow cytometer and assessing for red fluorescence intensity (PE-A). Staining with 1 μg/mL propidium iodide was conducted with the same incubation timepoints and was also assessed using the PE-A channel. For all flow cytometric analyses, the gating strategy used captured a minimum of 85% of the total events in all distinct treatments.

For upright microscopy with DCFH-DA- or MitoTracker Red-stained conidia, a small sample (~10 μL) of each treatment was taken before flow cytometric analysis and washed twice with 1× PBS (centrifugation at 2,500 × *g* for 5 minutes). Conidia were imaged by DIC microscopy on a Zeiss Axio Imager.MI microscope (Carl Zeiss) at 100× magnification, with the DsRed channel being used for MitoTracker Red and the eGFP channel being used for DCFH-DA.

### DCFH-DA staining in and flow cytometry with *A. fumigatus*, *C. albicans,* and *S. cerevisiae*

Conidia or yeast cells were prepared on the day of an experiment as described in “*In vitro* drug susceptibility with non-*Fusarium* species.” For *A. fumigatus*, conidia were resuspended in RPMI at a concentration of 2.5 × 10^5^ conidia/mL, split into 1 mL aliquots, and incubated at 37°C in the dark with 190 rpm for 2 hours. Compound treatments were then added to individual aliquots, and the conidia were returned to the incubator for 1 hour. After 1-hour incubation with the compound, each aliquot was treated with 1.25 μg/mL DCFH-DA and returned to the incubator for 30 minutes before analyzed on the flow cytometer (FITC-A). For *C. albicans* and *S. cerevisiae*, saturated overnight cultures in YPD were resuspended in 1 mL RPMI + AAs aliquots at a starting density of OD_600_ = 0.2. These aliquots were incubated at 30°C in the dark with 220 rpm for 3 hours, at which point compound treatments were added to individual cultures and returned to the incubator. After compound incubation, each aliquot was treated with 5 μg/mL DCFH-DA and returned to the incubator for 30 minutes before being run on the flow cytometer (FITC-A).

### Protoplasts generation and CRISPR-Cas9-mediated gene deletion in *F. graminearum*

To prepare an enzyme cocktail for cell wall digestion, 500 mg driselase (AA Blocks AA00G237), 200 mg yatalase (Takara T017), and 200 mg lysing enzyme (Sigma L3768) were added to 20 mL 1.2 M KCl in a small beaker and incubated at room temperature with stirring for 45 minutes. After incubation, the enzyme cocktail was passed through a 0.45-micron filter and placed on ice until required. To generate *F. graminearum* protoplasts, roughly 1–2 × 10^6^ *F. graminearum* conidia (strain Gz3639) were inoculated into 50 mL of Oxgall medium (per liter: 15 g Oxgall [Difco 212820], 10 g bactopeptone, 10 g D-glucose) in a 250 mL Erlenmeyer flask and incubated at 22°C and 200 rpm for 18 hours, or until the length of the germ tube was approximately 2–3 times the length of the conidium. At this point, germination was halted by placing the flask on ice. Germlings were isolated by passing the contents of the flask through a 0.4-micron filter and subsequently washed by passing 2 × 25 mL of chilled (4°C) ddH_2_O through the filter, followed by 25 mL of chilled 1.2 M KCl. Washed germlings were then gently transferred to a sterile petri plate or liquid reservoir and incorporated into the 20 mL chilled enzyme cocktail by gentle pipetting. The germling-enzyme cocktail mixture was then carefully transferred to a sterile 250 mL Erlenmeyer flask and incubated at 28°C, 100 rpm until roughly 70%–80% of the population was transformed into protoplasts, resembling circular yeast (anywhere between 1 hour and 90 minutes; after 1 hour, 10 μL aliquots were taken every 5–10 minutes and visualized with a light microscope to assess the progress of protoplast generation). Once this checkpoint was reached, the mixture was incubated on ice for 10 minutes to halt the activity of the enzyme cocktail. After incubation, the protoplasts were pelleted by centrifugation (4°C, 3,500 rpm, 5 minutes). The supernatant was removed, and protoplasts were washed by gently resuspending the pellet in 20 mL chilled 1.2 M KCl and centrifuging again. This process was repeated until the protoplasts had been washed with chilled 1.2 M KCl three times. Before the final centrifugation, a 10 μL aliquot of protoplasts was loaded onto a hemocytometer to calculate the total number of protoplasts present in the 20 mL mixture. Finally, the supernatant was once again removed after the final centrifugation, and the protoplasts were resuspended in an STC/DMSO solution (per mL: 930 μL STC buffer, 70 μL DMSO) to a final concentration of 1–2 × 10^7^ protoplasts/mL based on the hemocytometer counts (STC buffer recipe: 1.2M Sorbitol, 10 mM Tris-HCl, pH 8.0, 50 mM CaCl_2_). Protoplasts were separated into 200 μL aliquots in −80°C freezer-safe cryovials and stored at −80°C for long-term storage.

*F. graminearum yak1Δ* mutants were generated from Gz3639 protoplasts using a previously established CRISPR-Cas9-mediated protocol. CRISPR-Cas9 was set up using reagents and protocols from Integrated DNA Technologies (IDT), with guide RNAs (gRNAs) sequences selected using EuPaGDT (http://grna.ctegd.uga.edu/) and two gRNAs for each mutant (36). To produce 33 μM gRNAs, 33 μM of each CRISPR RNA (crRNA) was incubated with 33 μM of IDT’s generic trans-activating CRISPR RNA (tracrRNA; IDT 1072534) in IDT’s nuclease-free duplex buffer (IDT 11-01-03-01) for 5 minutes at 95°C and then cooled to room temperature for 10–15 minutes. These final concentrations were achieved by combining 5 μL of a 100 μM stock of each crRNA, 5 μL of a 100 μM stock of tracrRNA, and 5 μL of duplex buffer (final volume = 15 µL). Once cooled, RNP complexes were generated by taking each 1.5 μL of each gRNA and incubating alongside 0.75 μL of 1 μg/mL Cas9 nuclease (IDT 1081058) and 11 μL Cas9 working buffer (20 mM HEPES, 150 mM KCl, pH = 7.5), for a final volume of 13.25 μL. Cas9 nuclease was diluted from its stock concentration (10 μg/mL) to a concentration of 1 μg/mL in Cas9 working buffer. Each gRNA-Cas9 mixture was incubated at room temperature for 10 minutes, at which point the two gRNA-Cas9 mixtures corresponding to different guides for the same mutant were combined, creating a final reaction volume of 26.5 μL. This final reaction was incubated for another 5 minutes at room temperature, at which point it was added to an aliquot of Gz3639 protoplasts (thawed on ice from −80) alongside 6–8 μg repair template and 25 μL PEG 4000-CaCl_2_ buffer (60% wt/vol PEG 4000, 50 mM CaCl_2_, 450 mM, Tris-HCl, pH = 7.5). Briefly, three repair templates were generated via PCR (one for generating *yak1-1Δ*, one for generating *yak1-2Δ*, and one for generating a hygromycin-resistant strain without any gene disruption [WT_HygB_^R^]), using primers that amplified a hygromycin resistance (*hph*) cassette from the pRF-HU2 vector while introducing 50 bp of homology on either end to the 5′ and 3′ UTR of the *F. graminearum YAK1* locus (FGSG_05418). For the hygromycin-resistant strain without any gene disruption, the *hph* cassette was inserted in an intergenic region on Chromosome II (2905002-2916688). The entire transformation mixture was incubated on ice for 1 hour, at which point 1.5 mL of PEG 4000-CaCl_2_ buffer was added, and incubation continued at room temperature for 20 minutes. To initiate the recovery of protoplasts, the entire mixture was transferred to a sterile culture tube alongside 2 mL of STC buffer and 1.5 mL of liquid TB3 medium (recipe per liter: 3 g yeast extract, 3 g casamino acids, and 200 g sucrose) and incubated at 25°C with shaking (220 rpm) for 18–20 hours. An aliquot of this culture (300–1,000 μL) was then plated by mixing the aliquot with 20 mL of TB3 molten medium (liquid TB3 medium supplemented with 0.7% low melting point agarose [Chemscene CS-0182088], warmed to 42°C) supplemented with 100 μg/mL hygromycin B (BioShop Canada HYG003) in 50 mL falcon tube and then pouring this mixture onto a sterile petri plate. Plates were dried in a biosafety cabinet until the TB3 molten medium solidified, at which point they were transferred to 28°C and incubated until transformants were visible (2–5 days, depending on transformation efficiency). Putative transformants were confirmed by transferring a plug to PDA supplemented with 150 μg/mL hygromycin B and growing at 28°C for 2–3 days. Successful transformants were then cultured in CMC to generate conidia, at which point an individual conidium was isolated by plating a dilution of the CMC culture onto PDA supplemented with 150 μg/mL hygromycin B. An individual germling was then transferred to a fresh PDA + 150 µg/mL hygromycin B plate to grow, at which point gDNA was isolated with an E.Z.N.A. Plant DNA Kit (Omega Bio-Tek D3485-01) for genotyping. *yak1-1Δ and yak1-2Δ* mutants were genotyped by looking for both 5′ and 3′ integration, and absence of associated wild-type bands, while the WT_HygB_^R^ mutant was genotyped only for 5′ integration and absence of wild type. For additional information on crRNA sequences and genotyping primers ordered through IDT, refer to Table S3.

### *F. graminearum* pathology assays on mature wheat heads

Wheat (cv. Roblin) plants were grown in a growth chamber with a 16-hour photoperiod and a temperature cycle of 22°C (day) and 15°C (night). Once wheat heads were mid-anthesis (approximately 6–7 weeks post-germination), pathology assays were performed. In brief, wheat heads were dipped into a *F. graminearum* conidial suspension (at a concentration of 5 × 10^4^ conidia/mL) containing 0.01% Silwet L-77 and either compound treatments or an equivalent concentration of DMSO, until the heads were saturated. Inoculated heads were covered with a plastic bag and transferred to a contained misting facility. After 24 hours in the misting chamber, the plastic bags were removed, and after 48 hours, misting was turned off, and the plants were left to grow in the chamber. Plants were then monitored on a regular basis from 4 to 13 days for disease symptom development. Specifically, a disease score for each wheat head was calculated at each timepoint by dividing the number of diseased spikelets (i.e., either bleached or browned) by the total number of spikelets. The experiment was completed once wheat heads in the control treatment exhibited approximately 80%–90% infection (usually 11–13 days). Significance was assessed by a two-tailed t-test (adjusted via Benjamini-Hochberg), using the DMSO control for comparisons.

### *B. cinerea* spore preparation and detached tomato leaf infection

*Solanum lycopersicum* (cv. Glamour) plants were grown under 16 hours (22°C) of light and 8 hours (20°C) of darkness for 4 weeks before being used for infections. Eight distinct leaves across four distinct plants were used per treatment. *Botrytis cinerea* (strain MEE B191) was provided by the Canadian Collection of Fungal Cultures (Agriculture and Agri-Food Canada, Ottawa, ON, Canada). *B. cinerea* was grown for 1–2 weeks at room temperature on PDA to generate conidia. Conidia were taken from the plate using a cell scraper and transferred to a sterile 50 mL conical tube containing 10 mL Sabouraud Maltose Broth (SMB), pH = 5.6. The resulting mixture was then vortexed and passed through a cell strainer (100 μm pore size) to isolate conidia, which were then counted using a hemacytometer. The final concentration of conidia was then adjusted to 2.5 × 10^5^ conidia/mL using SMB, and individual aliquots were made to receive different compound treatments or a DMSO control. Ten microliters of the conidial suspension (containing compound treatments or DMSO control) was dropped on the midvein of detached leaves, after which the leaves were sealed in a petri dish with wet sterile filter paper to maintain high humidity. Plates were left under ambient light at room temperature for 3–4 days to allow for lesion development. Quantification of *B. cinerea* lesion size was done using Colour-analyzer (37).

### Rice growth conditions detached rice leaf infections with *M. oryzae*

Rice (*Oryza sativa*) cultivar CO-39 seedlings were used for the leaf spot inoculation assays. Seeds were germinated and grown in a controlled environment chamber at 28°C with a 12-hour light/dark cycle and 70% relative humidity. The wild-type strain Guy11 of *M. oryzae* was used for all experiments. Conidia were harvested from 10-day-old cultures grown on complete media (CM) agar plates at 25°C. The conidial suspension was prepared by flooding the plates with sterile distilled water and gently scraping the surface with a sterile spreader. The resulting suspension was filtered through sterile Miracloth, centrifuged at 10,000 RPM for 5 minutes, resuspended in sterile water, and adjusted to a concentration of 1 × 10^5^ conidia/mL using a hemocytometer.

Leaf spot infection assays were performed on 14-day-old rice seedlings. The conidial suspension was prepared in two concentrations of 1-CABC (50 µM and 200 µM) dissolved in sterile water. A 15 µL droplet of the prepared inoculum was carefully placed on the adaxial surface of a cut rice leaf mounted on 1% agarose. Inoculated leaves were incubated in a humid chamber at 25°C with a 12-hour light/dark cycle. Disease symptoms were evaluated 5 days post-inoculation by measuring the size of the lesions in ImageJ.

### Protoplast generation and CRISPR/Cas9-mediated genetic deletion in *M. oryzae*

Oligonucleotides used for deletion of *YAK1* in *M. oryzae* were designed with the aid of the Primer3 program (http://primer3.ut.ee/). Templates for sgRNA synthesis were designed with the aid of NEB’s online design tool (https://nebiocalculator.neb.com/#!/sgrna). All the oligonucleotides used in this study were purchased desalted from Merck Life Science and are listed in Table S3.

SgRNA for complexing with Cas9-NLS was synthesized using the EnGen sgRNA synthesis kit available from New England Biolabs (NEB #E3322), according to the instructions provided, and purified before complexing to Cas9 using the Monarch Spin RNA Cleanup Kit (50 µg) (NEB #T2040L). Cas9-NLS was purchased from New England Biolabs (NEB M0646M). Cas9-NLS was complexed with the sgRNA by a 10-minute incubation at room temperature (38). The sgRNA selection was carried out using the E-CRISP program at http://www.e-crisp.org/.

PEG-mediated fungal transformation of Glucanex-generated protoplasts was performed as previously described (39). Ribonucleoprotein (RNP) complexes and donor DNAs were introduced immediately prior to the step in the standard transformation procedure where 50% PEG is added, followed by incubation for 25 minutes. To ensure reproducibility, sgRNAs were freshly synthesized and purified on the day of transformation, and RNP complexes were kept on ice following assembly. Each transformation used 2 µg of donor DNA and 6 µg of Cas9 protein, mixed with the corresponding sgRNA at a 1:1 molar ratio and pre-incubated for 10 minutes at room temperature before the addition to 150 µL of protoplast suspension (1.5 × 10⁸ protoplasts/mL). For glufosinate selection, transformants were grown on Basta Defined Complex Medium (38). Glufosinate was added to the media at a final concentration of 40 µg/mL.

Genomic DNA from two Basta-resistant *yak1* mutants (*yak1-1* and *yak1-20*) was extracted using the Qiagen Plant DNeasy Plant Mini Kit (Qiagen # 69104) and sent to Novogene for whole-genome sequencing. Raw reads were aligned to the *M. oryzae* reference genome using samtools v1.9, before converting into BAM files and visualizing using IGV viewer, confirming complete deletion of the *YAK1* locus in both mutants.

### Ellman’s assay to assess the thiol-reactivity of 1-CABC

In a 96-well plate, DMSO, 1-CABC, and/or N-ethylmaleimide (NEM) were prepared at 2× the indicated concentrations, in either 50 μL phosphate buffer (per 100 mL: 0.259 g KH2PO4, 1.41 g K2HPO4, pH = 7.8) or 50 μL phosphate buffer supplemented with 200 μM NAC, and incubated for 5 minutes. To begin Ellman’s assay, 50 μL of 600 μM Ellman’s reagent (5,5′-dithiobis(2-nitrobenzoic acid); Sigma D8130) was added to all wells and mixed (final concentrations of 300 μM Ellman’s reagent and 100 μM NAC). The plate was then incubated for 20 minutes before reading absorbance at 412 nm in a plate reader. Absorbance values for compound treatments in the presence of Ellman’s reagent were corrected by subtracting absorbance values corresponding to phosphate buffer-only wells, and corrected values were then normalized to the absorbance of the DMSO control. Significance was assessed via a two-tailed t-test with a Holm correction, with comparisons made to the DMSO control.

## SUPPLEMENTAL MATERIAL

Supplemental material mbio.01291-26-s0001.pdf Supplemental figures and tables.

Table S3 mbio.01291-26-s0002.xlsx Primers used in this study.

ASM does not own the copyrights to Supplemental Material that may be linked to, or accessed through, an article. The authors have granted ASM a non-exclusive, world-wide license to publish the Supplemental Material files. Please contact the corresponding author directly for reuse.
