# Supplementary material for: A β-carboline natural product induces oxidative stress in Fusarium graminearum and limits the virulence of multiple fungal phytopathogens
Source: mBio. 2026 Jul 10;17(8):e01291-26. doi: 10.1128/mbio.01291-26 (PMC13463887; doi:10.1128/mbio.01291-26)
Supplement: Supplementary file 1 [file mbio.01291-26-s0001.pdf]

Supplemental Files

A β-carboline natural product induces oxidative stress in *Fusarium graminearum* and limits the virulence of multiple fungal phytopathogens

Bradley Laflamme[1], Christopher Blackman[2], Lauren Ryder[3], Mackenzie E.W. Loranger[4], Weibin Ma[3], Yunjin Lee[1], Margaret Balcerzak[2], Jessie MacAlpine[5], Luke Whitesell[1], Nicholas J. Talbot[3], Keiko Yoshioka[4], Rajagopal Subramaniam[2], Nicole Robbins[1], Leah E. Cowen[1,6]

[1]Department of Molecular Genetics, University of Toronto, Toronto, Ontario, M5S 1A8, Canada

[2]Agriculture and Agri-Food Canada, Ottawa, Ontario, K1A 0C6, Canada

[3]The Sainsbury Laboratory, University of East Anglia, Norwich Research Park, Norwich, NR4 7UH, UK

[4]Department of Cell and Systems Biology, University of Toronto, Toronto, Ontario, M5S 3B2, Canada

[5]Fungal Pathogenesis Section, Laboratory of Clinical Immunology and Microbiology, National Institute of Allergy & Infectious Diseases, National Institutes of Health, Bethesda, MD, 20892, United States

[6]Lead contact

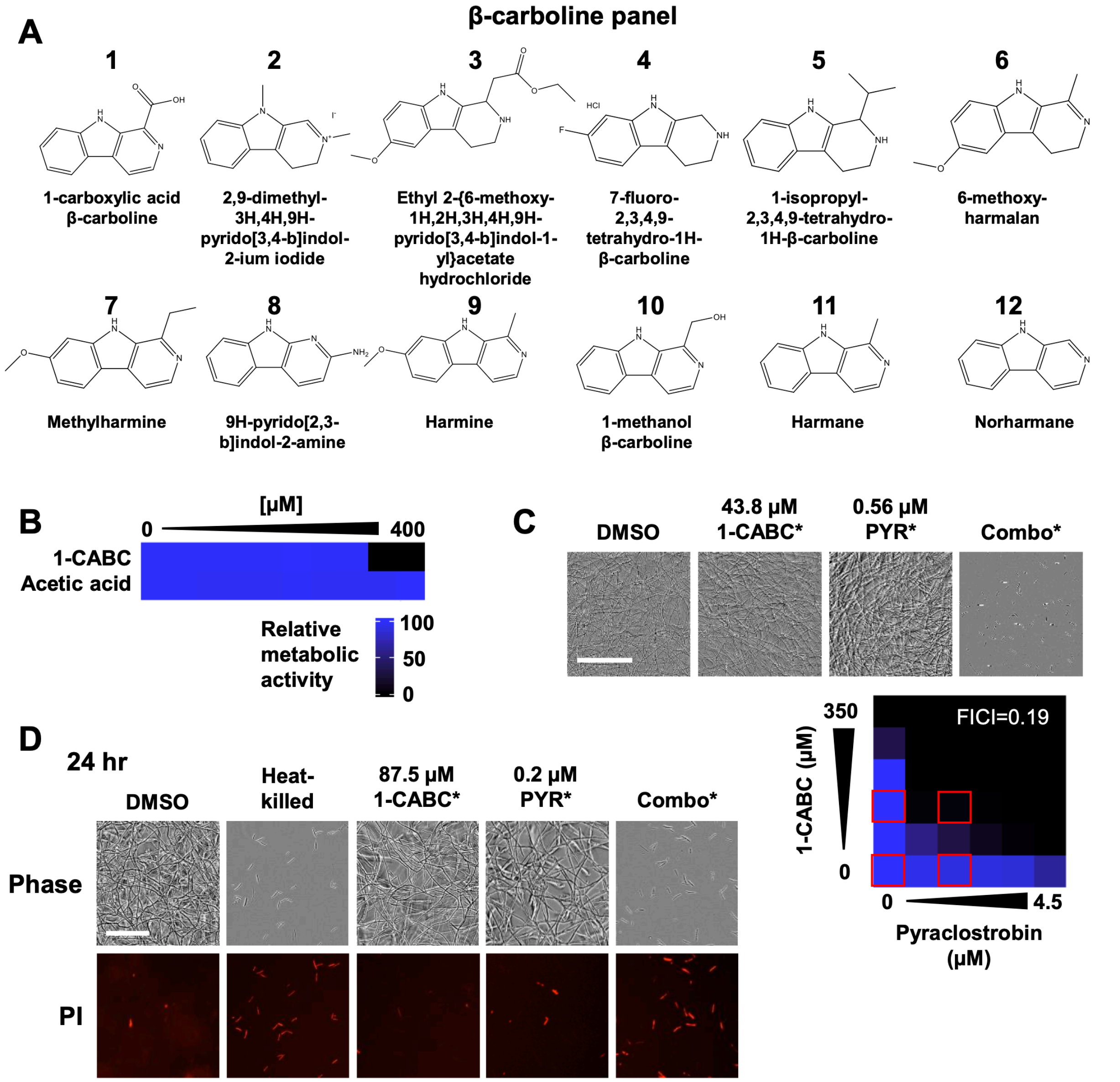

Supplemental Figure 1. **Supplemental information for Figure 1. A)** (1) 1-carboxylic acid β-carboline (1-CABC), (2) 2,9-dimethyl-3H,4H,9H-pyrido[3,4-b]indol-2-ium iodide, (3) ethyl 2-{6-methoxy-1H,2H,3H,4H,9H-pyrido[3,4-b]indol-1-yl}acetate hydrochloride, (4) 7-Fluoro-2,3,4,9-tetrahydro-1H-β-carboline, (5) 1-Isopropyl-2,3,4,9-tetrahydro-1H-β-carboline, (6) 6-methoxy-1-methyl-3H,4H,9H-pyrido[3,4-b]indole (6-Methoxyharmalan), (7) 1-ethyl-7-methoxy-9H-pyrido[3,4-b]indole (methylharmine), (8) 9H-pyrido[2,3-b]indol-2-amine, (9) harmine, (10) 1-methanol β-carboline, (11) harmane, (12) norharmane. See Supplemental Table 1 for information on chemical

distributors. **B)** An equimolar concentration of a simple carboxylic acid, acetic acid, does not have similar bioactivity to 1-CABC against *F. graminearum*. Dose-response assays were performed as in Figure 1A. **C)** Corresponding phase microscopy images highlighting the synergistic interaction between 1-CABC and PYR (Pyraclostrobin). The checkerboard assay (reproduced from Fig 1C below microscopy) is highlighted with red boxes to indicate the treatments shown in the microscopy image. Scale bar = 200 μm. **D)** 1-CABC displays fungicidal activity as monitored by propidium iodide (PI) uptake in combination with pyraclostrobin at 24 hr. Incucyte images were taken of *F. graminearum* conidia in the presence of the indicated compound treatments and 1 μg/ml PI using both the phase and red channel every hour for >30 hrs, as described for Figure 1D. Heat-killed conidia (95 °C for 10 min) were used as positive controls for PI uptake. Scale bar = 200 μm.

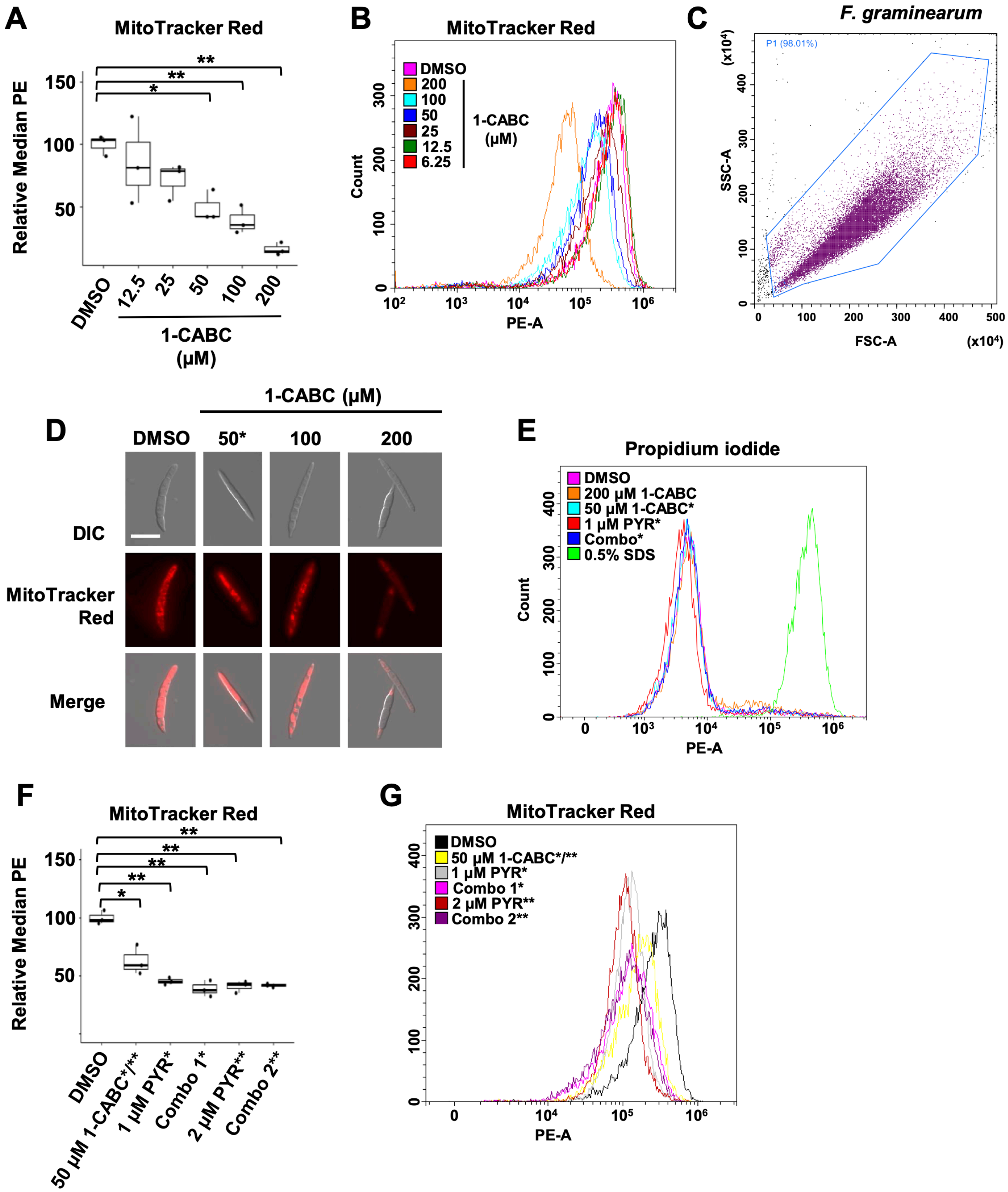
A
MitoTracker Red
Relative Median PE
DMSO
12.5
25
50
100
200
1-CABC (µM)
B
MitoTracker Red
DMSO
200
100
50
25
12.5
6.25
1-CABC (µM)
Count
PE-A
C
F. graminearum
P1 (98.01%)
SSC-A
FSC-A
D
1-CABC (µM)
DMSO
50*
100
200
DIC
MitoTracker Red
Merge
E
Propidium iodide
DMSO
200 µM 1-CABC
50 µM 1-CABC*
1 µM PYR*
Combo*
0.5% SDS
Count
PE-A
F
MitoTracker Red
Relative Median PE
DMSO
50 µM 1-CABC*/**
1 µM PYR*
Combo 1*
2 µM PYR**
Combo 2**
G
MitoTracker Red
DMSO
50 µM 1-CABC*/**
1 µM PYR*
Combo 1*
2 µM PYR**
Combo 2**
PE-A

Supplemental Figure 2. Supplemental data for Figure 3 on mitochondrial membrane potential. A)-B) 1-CABC decreases mitochondrial membrane potential in a dose-dependent manner as monitored by flow cytometry. **A)** Quantification of mitochondrial membrane potential via flow cytometry. The median red fluorescence (PE-A) values across 10,000 events for three distinct experiments were averaged and normalized as a percentage to the DMSO control to evaluate statistically significant deviations from DMSO fluorescence with a paired t-test (* = $p<0.05$; ** = $p<0.01$). **B)** A representative fluorescence histogram for Supplemental Figure 2A, showcasing the dose-dependent drop in membrane potential caused by 1-CABC. **C)** Gating strategy for Gz3639 conidia in flow cytometry based on forward (FSC-A) and side scatter area (SSC-A). **D)** Representative microscopy for *F. graminearum* conidia stained with MitoTracker Red. **E)** A 1-hr incubation with the indicated compound treatments does not kill *F. graminearum* conidia, as monitored by propidium iodide (PI) uptake. A fluorescence histogram showcases the red fluorescence intensity (PE-A) in the presence of the indicated compound treatments after PI exposure. 0.5% SDS was used as positive control for PI uptake via fungicidal activity. **F)** and **G)** 1-CABC and pyraclostrobin (PYR) do not lower mitochondrial membrane potential to a greater degree in combination. Experiments were conducted in the same manner as Supplemental Figures 2A and 2B.

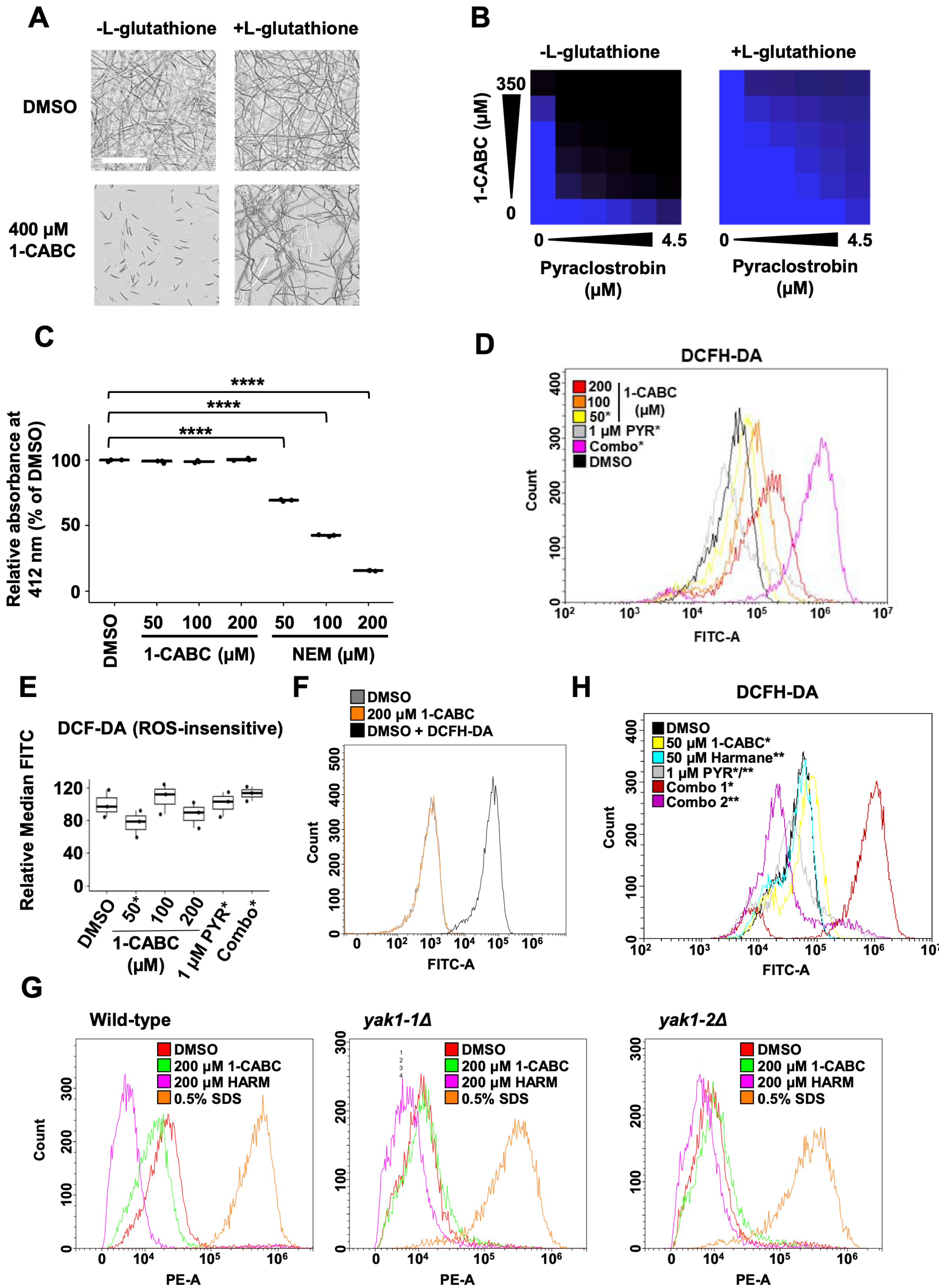
A
-L-glutathione
+L-glutathione
DMSO
400 µM
1-CABC
B
-L-glutathione
+L-glutathione
350
1-CABC (µM)
0
0
4.5
Pyraclostrobin
(µM)
C
****
****
****
Relative absorbance at
412 nm (% of DMSO)
100
50
0
DMSO
50
100
200
1-CABC (µM)
50
100
200
NEM (µM)
D
DCFH-DA
200
100
50*
1-CABC
(µM)
1 µM PYR*
Combo*
DMSO
Count
FITC-A
E
DCF-DA (ROS-insensitive)
Relative Median FITC
120
80
40
0
DMSO
50*
100
200
1-CABC
(µM)
1 µM PYR*
Combo*
F
DMSO
200 µM 1-CABC
DMSO + DCFH-DA
Count
FITC-A
H
DCFH-DA
DMSO
50 µM 1-CABC*
50 µM Harmane**
1 µM PYR*/**
Combo 1*
Combo 2**
Count
FITC-A
G
Wild-type
yak1-1Δ
yak1-2Δ
DMSO
200 µM 1-CABC
200 µM HARM
0.5% SDS
Count
PE-A

Supplemental Figure 3. Supplemental data for Figures 3 and 4 on intracellular ROS accumulation. A) Representative phase microscopy images of *F. graminearum* conidia after incubation with 400 μM 1-CABC in the presence or absence of 5 mM L-glutathione. Images were taken after a 24-hr incubation. Scale bar = 200 μm. **B)** Supplementation of L-glutathione dampens the interaction between 1-CABC and pyraclostrobin. Checkerboard assays were performed and visualized as in Figure 1C. **C)** 1-CABC does not exhibit thiol reactivity *in vitro* using Ellman's assay. The indicated treatments were incubated with 100 μM NAC for 5 minutes before being exposed to 300 μM Ellman's reagent, and absorbance at 412 nm was measured after 20 min incubation. Significance was assessed relative to the DMSO control (**** = $p<0.0001$). **D)** Corresponding fluorescence histogram for Figure 3F. **E)** Staining with the ROS-insensitive dye DCF-DA after drug treatments does not result in significant alterations to green fluorescence as measured by flow cytometry. Median FITC-A values were averaged from three distinct experiments and normalized as in Figure 3F. **F)** 1-CABC does not exhibit intrinsic fluorescence in *F. graminearum.* Conidia were treated with 1-CABC or DMSO in a manner similar to Figure 3E, only DCFH-DA was not added to the compound treatment. DMSO + DCFH-DA was also assayed to highlight the increase in fluorescence associated with DCFH-DA. **G)** Treatment of wild-type ($HygB^R$) or either *yak1Δ* mutant with 1-CABC or harmane (HARM) does not result in propidium iodide (PI) uptake as compared to 0.5% SDS. Assays were performed as in Supplemental Figure 2B.

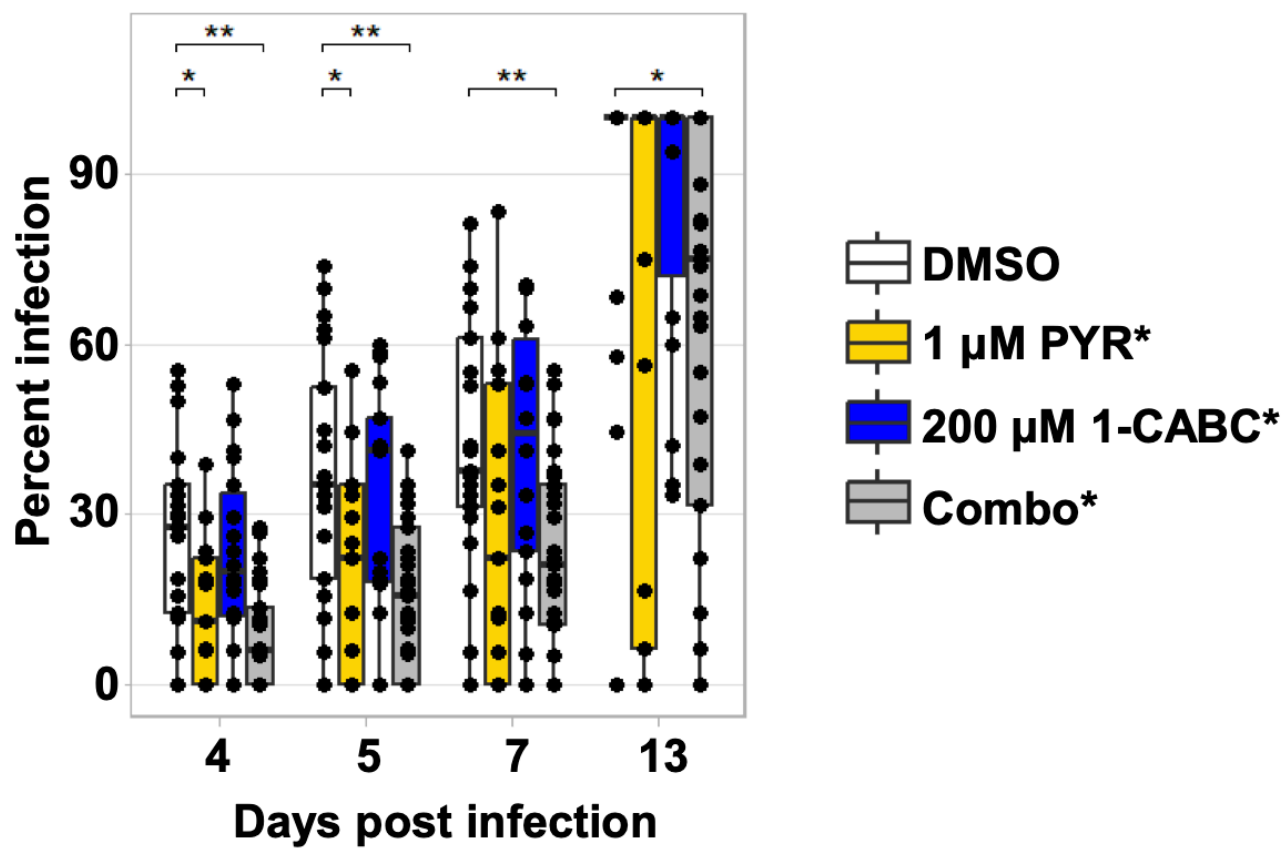

Supplemental Figure 4. Additional wheat head infection assay showcasing 1-CABC-pyraclostrobin synergy. Infections were conducted as in Figure 1D (* = $p<0.05$, ** = $p<0.01$).

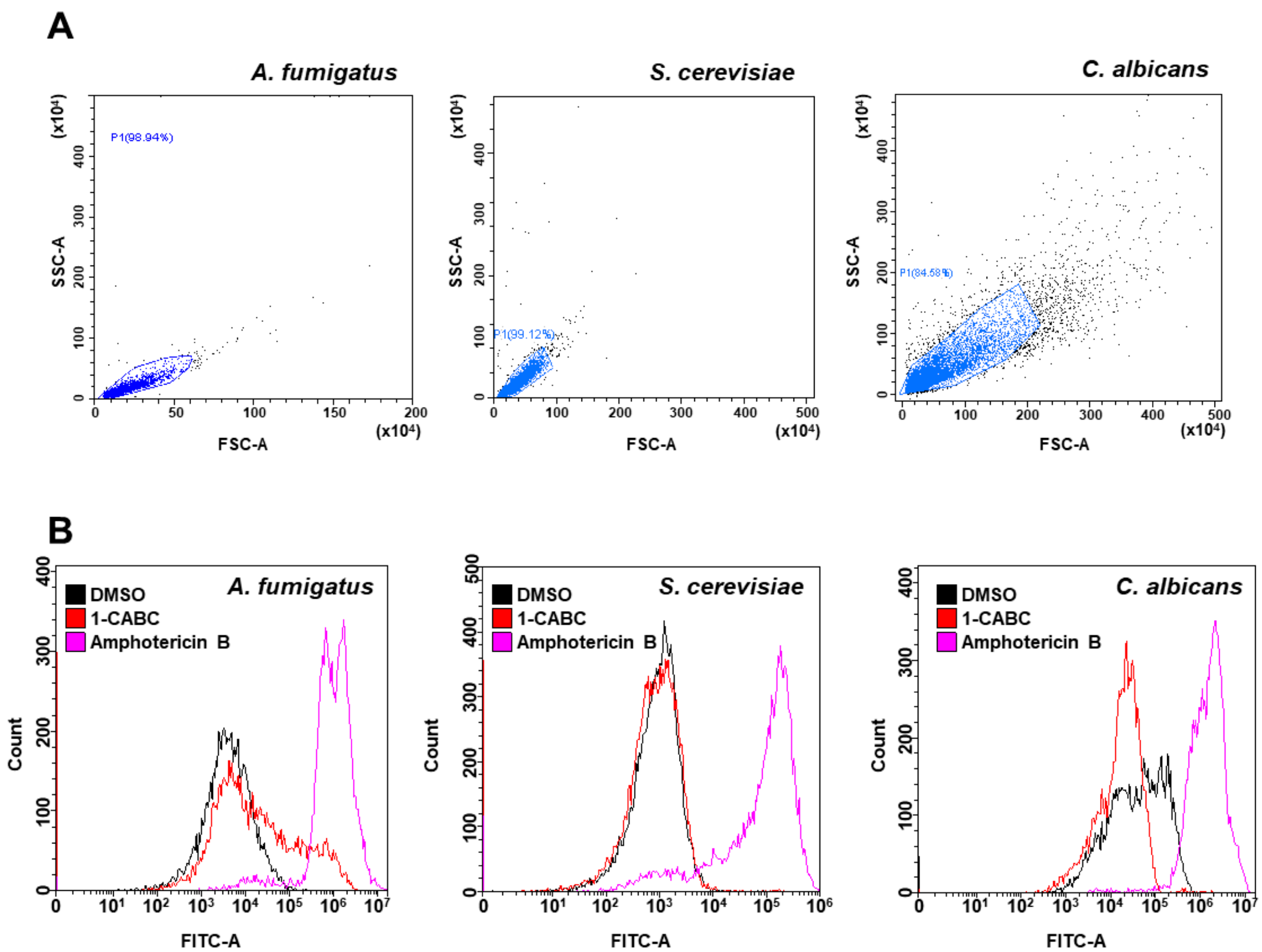

Supplemental Figure 5. Flow cytometry analysis of ROS induction by 1-CABC in distinct fungal species. A) Flow cytometry gating strategies for *A. fumigatus*, *S. cerevisiae*, and *C. albicans*. **B)** Corresponding flow cytometry plots for Figures 6C.

Supplemental Table 1. Commercially available β-carbolines used in Figure 1A.

β-carboline ID	Name	Distributor	Catalog_#
1	1-carboxylic acid β-carboline	Cayman	26052-96-0
2	2,9-dimethyl-3H,4H,9H-pyrido[3,4-b]indol-2-ium iodide	Sigma	S278165
3	ethyl 2-{6-methoxy-1H,2H,3H,4H,9H-pyrido[3,4-b]indol-1-yl}acetate hydrochloride	Sigma	R100935
4	7-Fluoro-2,3,4,9-tetrahydro-1H-β-carboline	Sigma	CDS021048
5	1-Isopropyl-2,3,4,9-tetrahydro-1H-β-carboline	Sigma	CDS020850
6	6-methoxy-1-methyl-3H,4H,9H-pyrido[3,4-b]indole (6-Methoxyharmalan)	Sigma	M1626
7	1-ethyl-7-methoxy-9H-pyrido[3,4-b]indole (methylharmine)	MolPort	MolPort-047-483-911
8	9H-pyrido[2,3-b]indol-2-amine	MolPort	MolPort-001-761-288
9	harmine	Sigma	286044
10	1-methanol β-carboline	MolPort	Molport-003-661-622
11	harmane	Sigma	103276
12	norharmane	Sigma	N6252

Supplemental Table 2. Fungal strains used in this manuscript.

Species	Strain ID	Genotype	Cowen ID
Aspergillus fumigatus	A1160	Wild-type	AfLC9065
Botrytis cinerea	MEE B191	Wild-type	BcLC10415
Candida albicans	CaSS1	Wild-type	CaLC6106
Fusarium graminearum	Gz3639	Wild-type	FgLC8588
Fusarium graminearum	Gz3639	*yak1-1Δ*	FgLC9403
Fusarium graminearum	Gz3639	*yak1-2Δ*	FgLC10386
Fusarium graminearum	Gz3639	*YAK1* (HygB-resistant)	FgLC9457
Saccharomyces cerevisiae	BY4741	Wild-type	ScLC151
Trichophyton indotineae	18	Wild-type	TiLC9502
Magnaporthe oryzae	Guy11	Wild-type	N/A
